# Mesenchymal stem cell-derived exosomal microRNA-182-5p alleviates myocardial ischemia/reperfusion injury by targeting GSDMD in mice

**DOI:** 10.1038/s41420-022-00909-6

**Published:** 2022-04-14

**Authors:** Rongchuan Yue, Shengzhong Lu, Yu Luo, Jing Zeng, Hao Liang, Dan Qin, Xiaobo Wang, Tao Wang, Jun Pu, Houxiang Hu

**Affiliations:** 1 Department of Cardiology, Affiliated Hospital of North Sichuan Medical College, Nanchong, 637000 P.R. China; 2 Cardiovascular Research Center, Affiliated Hospital of North Sichuan Medical College, Nanchong, 637000 P.R. China; 3 Academician Workstation, Affiliated Hospital of North Sichuan Medical College, Nanchong, 637000 P.R. China; 4 CSCSD, Affiliated Hospital of North Sichuan Medical College, Nanchong, 637000 P.R. China; 5 Department of Respiratory and Critical Care Medicine, University of Chinese Academy of Sciences Shenzhen Hospital, Shenzhen, 518100 P.R. China

**Keywords:** Biotechnology, Cardiovascular diseases

## Abstract

Recent evidence indicates that exosomes derived from mesenchymal stem cells (MSCs) confer protective effects against myocardial ischemia/reperfusion (I/R) injury. Exosomes are carriers of potentially protective endogenous molecules, including microRNAs (miRNAs/miRs). The current study set out to test the effects of transferring miR-182-5p from MSC-derived exosomes into myocardial cells on myocardial I/R injury. First, an I/R mouse model was developed by left anterior descending coronary artery occlusion, and myocardial cells were exposed to hypoxia/reoxygenation (H/R) for in vitro I/R model establishment. Loss- and gain-of-function experiments of miR-182-5p and GSDMD were conducted to explore the effects of miR-182-5p via MSC-derived exosomes on cell pyroptosis and viability. GSDMD was robustly expressed in I/R-injured myocardial tissues and H/R-exposed myocardial cells. GSDMD upregulation promoted H/R-induced myocardial cell pyroptosis and reduced viability, corresponding to increased lactate dehydrogenase release, reactive oxygen species production, and pyroptosis. A luciferase assay demonstrated GSDMD as a target of miR-182-5p. In addition, exosomal miR-182-5p was found to diminish GSDMD-dependent cell pyroptosis and inflammation induced by H/R. Furthermore, MSC-derived exosomes carrying miR-182-5p improved cardiac function and reduced myocardial infarction, accompanied with reduced inflammation and cell pyroptosis in vivo. Taken together, our findings suggest a cardioprotective effect of exosomal miR-182-5p against myocardial I/R injury, shedding light on an attractive therapeutic strategy.

## Introduction

Myocardial ischemia/reperfusion (I/R) injury ensues secondary to a resumption of blood flow in the ischemic myocardium following the severe and abrupt obstruction of blood flow [1]. Myocardial I/R results in activation of nucleotide-binding oligomerization domain-like receptor family pyrin domain-containing 3 (NLRP3) inflammasome, along with the augmentation of inflammation and myocardial cell death via the process of caspase-1-dependent pyroptosis [2]. Moreover, excessive release of toxic reactive oxygen species (ROS) in the re-perfused myocardium disrupts redox homeostasis, which is known to evoke cellular stress in response to I/R [3]. Currently, tackling post-infarction heart failure represents an effective treatment modality and a feasible strategy for preventing myocardial I/R injury-associated death [4]. Furthermore, mesenchymal stem cell (MSC)-based therapy is recently highlighted as a striking option for treating inflammatory and cardiovascular diseases [5]. Accumulating preclinical evidence further indicates that MSC-derived extracellular vesicles, such as exosomes, hold potential in the treatment of myocardial I/R damage, however, the underlying therapeutic mechanisms remain elusive [6, 7]. On the other hand, the crucial functions of exosomes originated from diverse types of stem cells encompassing pluripotent stem cells, cardiac progenitor cells, embryonic stem cells, and MSCs have been extensively reported in the mediation of cardiac functions. More specifically, these exosomes have been illustrated to confer anti-apoptotic effects on both hosts as well as transplanted cells, and to exert pro-angiogenic actions, thus reducing infarct size and accelerating cardiac recovery [8–11]. Inherently, exosomes can carry and transport proteins and RNAs, including microRNAs (miRNAs or miRs), while their uptake during cell-to-cell communication alters the function and behaviors of recipient cells [12]. Recently, exosomes from several different sources have also been shown to carry cardioprotective miRNAs against myocardial I/R damage in animal models [13–15]. For instance, a previous study found that exosomal miR-181b from cardiosphere-derived cells (CDCs) into macrophages conferred the cardioprotective effects of CDC administration after reperfusion [16]. In the light of the above studies, it would be plausible to suggest that the identification of novel exosomal miRNAs may aid in the development of stem cell-based therapy for attenuating myocardial I/R damage.

Being widely-researched, miRNAs are known as endogenous non-coding ~22-nucleotide RNAs that mediate translational repression of complementary messenger RNAs (mRNAs) by forming and directing RNA-induced silencing complex against target mRNAs [17]. There is sound evidence suggesting that miRNAs exert regulatory functions on gene expression-mediated events in normal cardiac development and in various cardiovascular disorders, such as coronary artery disease and myocardial infarction (MI) [18]. For example, one such miRNA, miR-21 was previously highlighted to play a cardioprotective role in mice with MI [19]. Moreover, delivery of exosomal miR-21 by MSCs can also ameliorate MI-induced myocardial damage [9]. Meanwhile, another miRNA miR-182-5p is poorly-expressed in H9c2 myocardial cells under hypoxic conditions [20]. Also, treatment with a small molecular weight miR-182 inducer protects against I/R injury by inhibiting myocardial cell death [21]. However, it remains to be determined whether exosomal miR-182-5p has inherent cardioprotective effects.

Gasdermin D (GSDMD) is a 487 amino acid cytoplasmic protein with roles in epithelial differentiation and tumor suppression, and may also be involved in myocardial I/R injury via its activation by inflammasome to cause pyroptosis [22, 23]. Moreover, GSDMD is known to trigger the secretion of the pro-inflammatory cytokine caspase-1 in sepsis-induced endothelial cell injury [24]. Initial bioinformatics analyses in our study indicated the presence of a functional miR-182-5p binding site in the 3′-untranslated region (3′UTR) of GSDMD mRNA. Consequently, we proposed a hypothesis that miR-182-5p may mediate I/R-associated myocardial damage through the regulation of GSDMD, and performed a battery of experiments to ascertain the function and potential mechanism of the miR-182-5p/GSDMD axis participating in the myocardial cell pyroptosis in the course of myocardial I/R injury.

## Results

### I/R induces myocardial cell injury and pyroptosis in myocardial tissues

**Fig. 1:**
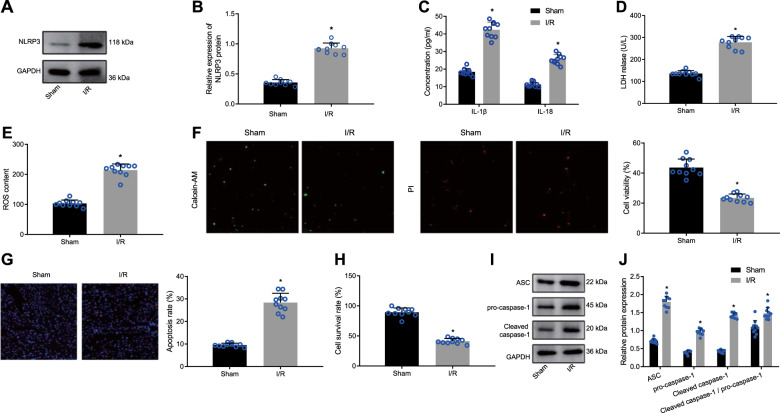
I/R induces myocardial cell injury and pyroptosis in the mouse myocardial tissues. **A** Representative images of NLRP3 protein bands in myocardial tissues of mice; **B** Western blot analysis of NLRP3 protein in myocardial tissues of mice, normalized to GAPDH; **C** The concentration of IL-1β and IL-18 in myocardial tissue homogenate supernatant of mice determined by ELISA; **D** LDH release in myocardial tissues of mice detected by LDH kit. **E** ROS content in myocardial tissues of mice evaluated by DCFH-DA; **F** The cell viability in myocardial tissues of mice measured by Calcein-AM/PI double staining; **G** The TUNEL-positive apoptotic cells (red) in myocardial tissues of mice; **H** The cell survival rate in myocardial tissues of mice measured by trypan blue staining; **I** Representative images of ASC, pro-caspase-1, and caspase-1 protein bands in myocardial tissues of mice; **J** Western blot analysis of ASC, pro-caspase-1, and caspase-1 proteins in myocardial tissues of mice, normalized to GAPDH; *N* = 10 for mice in each group. Measurement data were expressed as mean ± standard deviation. Data comparison between two groups was conducted by unpaired *t*-test. *P < 0.05 vs. sham-operated mice.

Existing research has shown that reoxygenation following ischemia causes tissue oxidative stress and that the ROS-induced NLRP3 inflammasome can aggravate myocardial I/R injury by initiating pyroptosis and sterile inflammatory responses [25]. To establish a mouse model of I/R, the left anterior descending (LAD) coronary artery was occluded, followed by reperfusion. After isolation of the myocardial tissues and cells from I/R-rendered mice, we evaluated the alterations in NLRP3, IL-1β, IL-18, ROS, pyroptosis, and lactate dehydrogenase (LDH) activity in response to I/R. Western blot analysis results revealed an increased NLRP3 protein level in the myocardial tissues of I/R-rendered mice (Fig. 1A, B). In addition, enzyme-linked immunosorbent assay (ELISA) data demonstrated that the IL-1β and IL-18 concentration was elevated in the myocardial tissue homogenate supernatant of I/R-operated mice (Fig. 1C), thus indicative of active NLRP3 inflammasome.

As I/R can induce tissue oxidative stress and LDH activity change [26, 27], we measured the ROS content and LDH activity in the myocardial tissues, the results of which exhibited significant increases in the ROS content and LDH activity in the myocardial tissues of I/R mice (Fig. 1D, E). Existing studies have shown that NLRP3 inflammasome activation can elicit cell pyroptosis [28] and apoptosis [29]. Therefore, we subsequently assessed the viability, apoptosis, and survival rate of myocardial cells using Calcein-AM/PI double staining, TUNEL staining, and trypan blue staining, respectively. The results showed suppressed cell viability and survival rate and enhanced apoptosis (Fig. 1F–H) in the myocardial tissues in response to I/R. In addition, we measured the alterations in pyroptosis-related proteins. Expectedly, the protein levels of ASC, pro-caspase-1, and cleaved caspase-1, as well as cleaved caspase-1/pro-caspase-1 ratio, were all remarkably elevated in the myocardial tissues from I/R mice (Fig. 1I, J). Altogether, these results suggested that I/R could stimulate myocardial cell injury and pyroptosis in myocardial tissues.

### GSDMD expression is upregulated in I/R-injured myocardial tissues

**Fig. 2:**
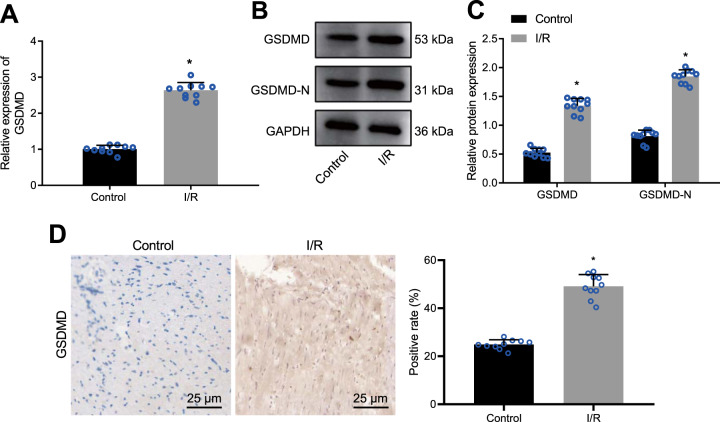
GSDMD is robustly expressed in myocardial tissues of I/R mice. **A** mRNA expression of GSDMD in myocardial tissues of mice with I/R detected by RT-qPCR, normalized to GAPDH; **B** Representative images of GSDMD and GSDMD N protein bands in myocardial tissues of mice with I/R. **C** Western blot analysis of GSDMD protein and GSDMD N in myocardial tissues of mice with I/R, normalized to GAPDH; **D** Immunohistochemistry staining of GSDMD protein in myocardial tissues of mice with I/R. *N* = 10 for mice in each group. Measurement data were expressed as mean ± standard deviation. Data comparison between two groups was conducted by unpaired *t*-test. *p < 0.05 vs. sham-operated mice.

GSDMD is the ligand-protein involved in NLRP3 inflammasome-induced cell pyroptosis [30], which consists of GSDMD N and C domains and can be cleaved by inflammatory caspases to induce pyroptosis, wherein GSDMD N triggers higher pyroptosis. Here, we determined the expression patterns of GSDMD and N-terminus of GSDMD in the I/R-injured myocardial tissues by reverse transcription-quantitative polymerase chain reaction (RT-qPCR) and Western blot analysis. The results revealed that GSDMD mRNA (Fig. 2A) and protein levels (Fig. 2B, C) of GSDMD and GSDMD N were elevated in myocardial tissues of mice with I/R. In addition, immunohistochemical staining results also demonstrated a high positive expression of GSDMD protein in the mouse model of I/R (Fig. 2D). Thus, GSDMD expression was augmented in the myocardial tissues of mice with I/R.

### GSDMD increases H/R-induced myocardial cell pyroptosis and injury

**Fig. 3:**
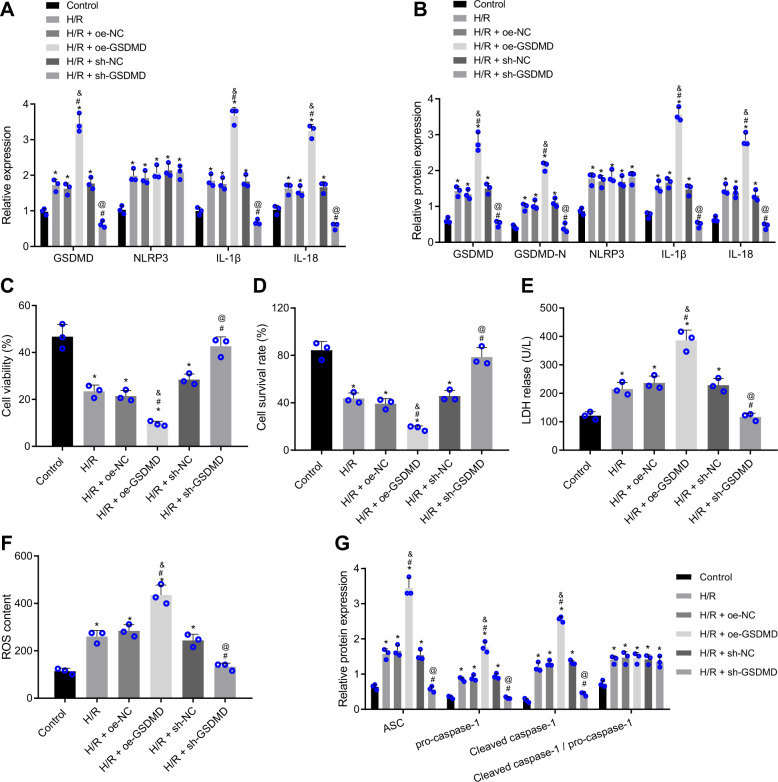
GSDMD induces H/R-induced myocardial cell pyroptosis and injury. **A** The relative mRNA expression of GSDMD, NLRP3, IL-1β, and IL-18 in H/R-exposed myocardial cells detected by RT-qPCR; **B** Western blot analysis of GSDMD, NLRP3, IL-1β, IL-18, and GSDMD N proteins in H/R-exposed myocardial cells, normalized to GAPDH; **C** The viability of H/R-exposed myocardial cells assessed by Calcein-AM/PI double staining. **D** The H/R-exposed myocardial cell survival rate measured by trypan blue staining; **E** LDH release in H/R-exposed myocardial cells detected by LDH kit; **F** ROS production in H/R-exposed myocardial cells stained with DCFH-DA; **G** Western blot analysis of ASC, pro-caspase-1, and caspase-1 proteins as well as a cleaved caspase-1/pro-caspase-1 ratio in H/R-exposed myocardial cells, normalized to GAPDH. Measurement data were expressed as mean ± standard deviation. The comparison among multiple groups was conducted with one-way ANOVA, followed by Tukey’s post hoc test. Each cell experiment was repeated three times independently. *P < 0.05 vs. untreated myocardial cells, #*P* < 0.05 vs. H/R-exposed myocardial cells, &*P* < 0.05 vs. H/R-exposed myocardial cells transfected with oe-NC, @ *P* < 0.05 vs. H/R myocardial cells transfected with sh-NC.

Primary myocardial cells were isolated and cultured in vitro. The immunofluorescence staining for detection of cardiac troponin I (cTnI) indicated that the purity of isolated myocardial cells reached above 95% (Supplementary Fig. 1). The role of GSDMD was then assessed in the H/R-induced cell models with GSDMD gain- and loss-of-function studies. Based on the RT-qPCR and Western blot data, the mRNA and protein levels of GSDMD, GSDMD N, NLRP3, IL-1β, and IL-18 were determined to be upregulated in the H/R-exposed myocardial cells while treatment with sh-GSDMD reduced those of GSDMD, GSDMD N, IL-1β, and IL-18 without altering that of NLRP3. In contrast, H/R-exposed myocardial cells treated with oe-GSDMD exhibited elevations in the mRNA and protein levels of GSDMD, GSDMD N, IL-1β, and IL-18 without changes in that of NLRP3 (Fig. 3A, B). These data indicate that GSDMD may be involved in the release of IL-1β and IL-18 in the inflammasome of NLRP3.

In response to microbial infection and danger signals, activated caspases can cleave GSDMD to form GSDMD N, which can induce inflammatory death (heat-shock) and release of various inflammatory cytokines [31]. Belnacasan (VX-765; 10 μm) was adopted to treat myocardial cells for 24 h to block the generation of GSDMD N from GSDMD induced by activated caspases. The results of Western blot analysis showed that the expression patterns of cleaved caspase-1, GSDMD N, IL-1β, and IL-18 in the H/R-exposed myocardial cells were lowered upon treatment with VX-765 (Supplementary Fig. 2).

Moreover, we observed reduced cell viability (Fig. 3C) and survival rate (Fig. 3D), elevated LDH activity (Fig. 3E), and ROS production (Fig. 3F), enhanced protein levels of ASC, pro-caspase-1, and caspase-1, along with an increased cleaved caspase-1/pro-caspase-1 ratio (Fig. 3G) in the myocardial cells after H/R exposure. Upon depletion of GSDMD in the H/R-exposed myocardial cells, significant increases were evident in the cell viability and survival rate, yet reductions in the LDH level, ROS production, and ASC, pro-caspase-1, and caspase-1 expression. When GSDMD was overexpressed, contrasting results were obtained. These results showed that GSDMD could stimulate H/R-induced myocardial cell pyroptosis and injury.

### GSDMD is a target gene of miR-182-5p

**Fig. 4:**
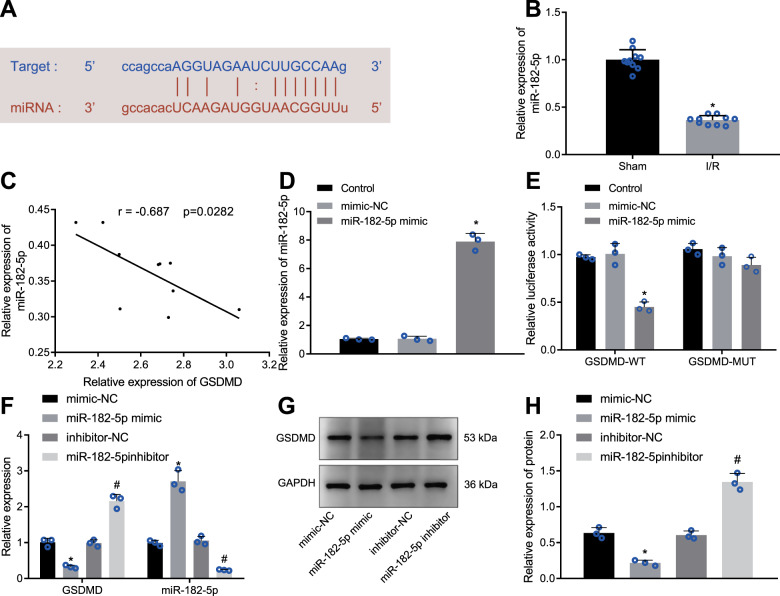
miR-182-5p targets GSDMD. **A** miR-182-5p putative binding sites in GSDMD 3′UTR predicted by starBase; **B** The relative expression of miR-182-5p in I/R-injured myocardial tissues detected by RT-qPCR, normalized to U6; **C** The correlation of miR-182-5p expression with GSDMD expression in I/R-injured myocardial tissues; **D** The miR-182-5p expression in myocardial cells determined by RT-qPCR; **E** The binding of miR-182-5p to GSDMD in HEK-293T cells confirmed by dual-luciferase reporter assay; **F** The miR-182-5p expression and relative mRNA expression of GSDMD in myocardial cells detected by RT-qPCR; **G** Representative images of GSDMD protein bands; **H** Western blot analysis of GSDMD protein in myocardial cells, normalized to GAPDH. *N* = 10 for mice in each group. Measurement data were expressed as mean ± standard deviation. The comparison between two groups was conducted by unpaired *t*-test. Each cell experiment was repeated three times independently. *P < 0.05 vs. sham-operated mice or HEK-293T cells or myocardial cells transfected with mimic-NC, #*P* < 0.05 vs. myocardial cells transfected with inhibitor-NC.

Existing research has reported the potential of miR-182-5p to inhibit myocardial I/R injury [20, 21], yet there is no evidence supporting its role in cell pyroptosis following myocardial I/R. Therefore, we attempted to elucidate the downstream mechanism by which miR-182-5p might regulate the cell pyroptosis in myocardial I/R. Initially, a binding site between mmu-miR-182-5p and GSDMD 3’UTR was predicted by StarBase (Fig. 4A). Next, we conducted RT-qPCR for RNA quantitation and our results (Fig. 4B) exhibited poor miR-182-5p expression in the I/R-injured mouse myocardial tissues. In addition, Pearson’s correlation analysis uncovered an inverse relation between miR-182-5p expression and GSDMD expression in the I/R-injured myocardial tissues (Fig. 4C). Then, we achieved an enhancement in the miR-182-5p expression in myocardial cells by manipulation with miR-182-5p mimic (Fig. 4D). Luciferase assay offered data demonstrating miR-182-5p’s specific binding to GSDMD that miR-182-5p enhancement remarkably reduced the luciferase activity of GSDMD-wild type (WT) in HEK-293T cells without altering that of GSDMD-mutant type (MUT) (Fig. 4E). In addition, as illustrated in Fig. 4F-H, the mRNA and protein levels of GSDMD were elevated in the myocardial cells upon depletion of miR-182-5p, while its upregulation annulled this effect. Therefore, miR-182-5p could target GSDMD and negatively regulate its expression.

### Successful isolation of MSCs and MSC-derived exosomes

MSCs were separated from the bone marrow of C57BL/6 mice. The isolated MSCs were observed to adhere three days postinoculation. The adherent MSCs were spindle-shaped, and grew in a spiral or clustered fashion, showing clear nuclei (Supplementary Fig. 3A). To detect the surface markers of MSCs for further characterization of isolated MSCs, flow cytometry was performed. As shown in Supplementary Fig. 3B, the adherent MSCs were positive for CD105, CD73, and CD90, but negative for CD45, CD34, and CD11b. A series of staining assays supported the osteogenic, adipogenic, and chondrogenic differentiating potentials of the MSCs (Supplementary Fig. 3C).

We then characterized the MSC-derived exosomes by transmission electron microscope (TEM) and nanoparticle tracking analysis (NTA), which showed their diameter ranged within 30–120 nm, and characteristics of round-shaped or elliptical-shaped membranous vesicles (Supplementary Fig. 3D, E). Additionally, the protein levels of CD63, HSP70, TSG101, and Alix were upregulated yet Calnexin was absent in the MSC-derived exosomes (Supplementary Fig. 3F). Flow cytometric data revealed that MSC-derived exosomes were CD63-positive (Supplementary Fig. 3G). Collectively, these data demonstrated the successful isolation of MSCs and MSC-derived exosomes.

### miR-182-5p delivered by MSC-derived exosomes reduces GSDMD expression in myocardial cells

**Fig. 5:**
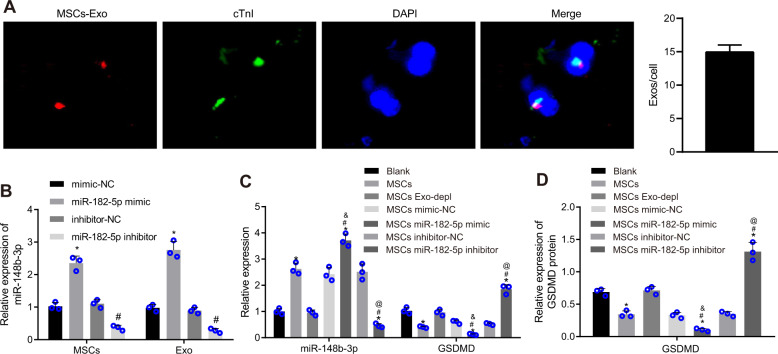
Exosomal miR-182-5p from MSCs reduces GSDMD expression in myocardial cells. **A** The uptake of exosomes by myocardial cells observed under an inverted microscope (× 200); **B** The relative expression of miR-182-5p in MSCs and MSC-derived exosomes determined by RT-qPCR, normalized to U6; *P < 0.05 vs. the MSCs transfected with mimic-NC or their derived exosomes; #*P* < 0.05 vs. the MSCs transfected with inhibitor-NC or their derived exosomes. **C** miR-182-5p expression and GSDMD mRNA level in myocardial cells co-cultured with miR-182-5p mimic/inhibitor-transfected MSCs detected by RT-qPCR, normalized to U6 and GAPDH, respectively; **D** Western blot analysis of GSDMD protein in myocardial cells co-cultured with miR-182-5p mimic/inhibitor-transfected MSCs, normalized to GAPDH. Measurement data were expressed as mean ± standard deviation. The comparison between two groups was performed using an unpaired *t*-test and comparison among multiple groups was assessed with one-way ANOVA, followed by Tukey’s post hoc test. Each cell experiment was repeated three times independently. *P < 0.05 vs. the blank myocardial cells, #*P* < 0.05 vs. myocardial cells co-cultured with non-transfected MSCs, ^&^*P* < 0.05 vs. myocardial cells co-cultured with mimic-NC-transfected MSCs, @ *P* < 0.05 vs. myocardial cells co-cultured with inhibitor-NC-transfected MSCs.

PKH26-stained exosomes were co-incubated together with myocardial cells, and the red fluorescence signal was evident in the myocardial cells under a confocal fluorescence microscope (Fig. 5A), suggesting that the myocardial cells can internalize the PKH26-labeled exosomes. To determine whether MSC-derived exosomes could deliver miR-182-5p to the myocardial cells, MSCs were introduced with miR-182-5p mimic or inhibitor before exosome extraction. RT-qPCR results indicated that either the MSCs manipulated with miR-182-5p mimic or the derived exosomes exhibited an elevation in the miR-182-5p expression, which was contrasting in both the MSCs transfected with miR-182-5p inhibitor and the derived exosomes (Fig. 5B).

Next, we utilized GW4869 to treat MSCs to block the secretion of exosomes and then co-cultured MSCs with myocardial cells. RT-qPCR and Western blot analysis results illustrated that GSDMD expression decreased while that of miR-182-5p was elevated in the myocardial cells co-cultured with untreated MSCs. The expression of miR-182-5p and GSDMD in myocardial cells was unchanged following co-culture with GW4869-treated MSCs. After exposure to miR-182-5p mimic-treated MSCs, the myocardial cells presented with reduced GSDMD expression along with elevated miR-182-5p expression. Conversely, co-incubation with miR-182-5p inhibitor-treated MSCs led to opposite results in myocardial cells (Fig. 5C, D). Cumulatively, delivery of miR-182-5p via MSC-released exosomes effectively repressed the GSDMD expression in myocardial cells.

### miR-182-5p delivered by MSC-derived exosomes reduces GSDMD expression to inhibit H/R-induced pyroptosis and injury of myocardial cells

**Fig. 6:**
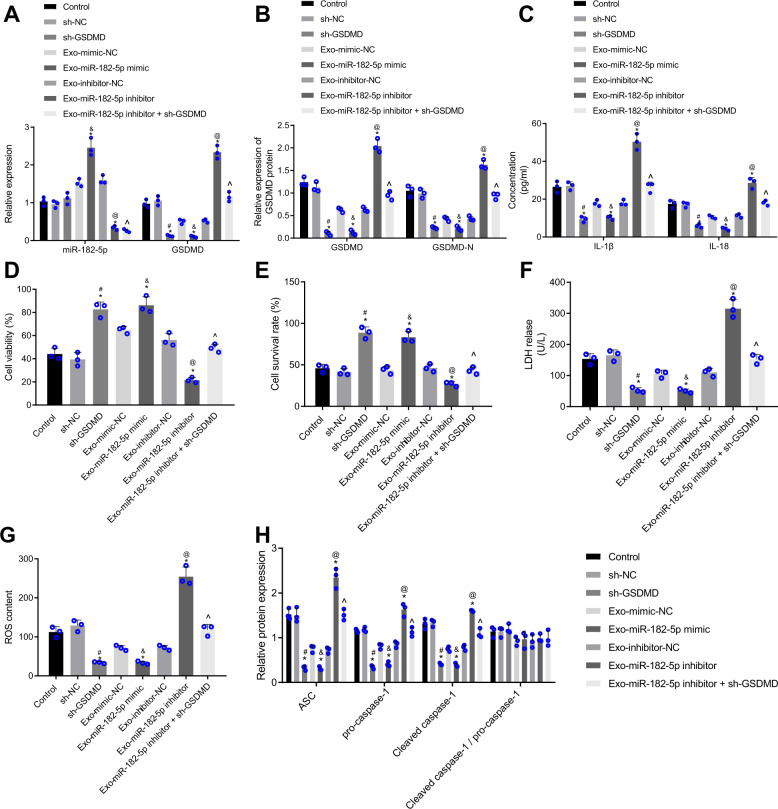
Exosomal miR-182-5p from MSCs inhibits inflammation and pyroptosis of H/R-exposed myocardial cells via inhibition of GSDMD. H/R-exposed myocardial cells were co-cultured with MSC-derived exosomes or not. GSDMD expression was silenced in H/R-exposed myocardial cells while miR-182-5p expression was altered in MSC-derived exosomes using mimics or inhibitors before co-culture. **A** miR-182-5p expression and GSDMD mRNA level in H/R-exposed myocardial cells detected by RT-qPCR, normalized to U6 and GAPDH, respectively; **B** Western blot analysis of GSDMD protein and GSDMD N in H/R-exposed myocardial cells, normalized to GAPDH; **C** The levels of IL-1β and IL-18 in the supernatant of H/R-exposed myocardial cells determined by ELISA; **D** The viability of H/R-exposed myocardial cells assessed by Calcein-AM/PI double staining; **E** The survival rate of H/R-exposed myocardial cells measured by trypan blue staining; **F** LDH release in H/R-exposed myocardial cells detected by LDH kit; **G** ROS production in H/R-exposed myocardial cells examined by DCFH-DA; **H** Western blot analysis of ASC, pro-caspase-1, and caspase-1 proteins as well as the cleaved caspase-1/pro-caspase-1 ratio in H/R-exposed myocardial cells, normalized to GAPDH. Measurement data were expressed as mean ± standard deviation. The comparison among multiple groups was conducted with one-way ANOVA, followed by Tukey’s post hoc test. Each cell experiment was repeated three times independently. *P < 0.05 vs. H/R-exposed myocardial cells without transfection, #*P* < 0.05 vs. H/R-exposed myocardial cells transfected with sh-NC and co-cultured with exosomes, &*P* < 0.05 vs. H/R-exposed myocardial cells co-cultured with exo-mimic-NC, @*P* < 0.05 vs. H/R-exposed myocardial cells co-cultured with exo-inhibitor-NC, ^*P* < 0.05 vs. H/R-exposed myocardial cells transfected with sh-GSDMD.

The aforementioned results showed that MSCs could transfer miR-182-5p into the myocardial cells via exosomes. We then sought to ascertain the modulatory function of MSC-derived exosomes in H/R-induced myocardial cell injury. The exosomes sourced from miR-182-5p-abundant or -deficient MSCs (exo-miR-182-5p mimic or exo-miR-182-5p inhibitor) were co-cultured with the H/R-exposed myocardial cells. The RT-qPCR and Western blot results displayed in Fig. 6A, B showed that the sh-GSDMD markedly decreased the GSDMD and GSDMD N expression in H/R-exposed myocardial cells without affecting miR-182-5p. miR-182-5p expression was elevated when myocardial cells were co-incubated with exo-miR-182-5p mimic, accompanied with loss of GSDMD and GSDMD. The reductions in GSDMD and GSDMD N levels induced by sh-GSDMD could be restored after miR-182-5p inhibition by co-culture with exo-miR-182-5p inhibitor.

As depicted by ELISA data (Fig. 6C), knockdown of GSDMD or the upregulation of miR-182-5p delivered by exo-miR-182-5p mimic contributed to the reduced levels of IL-1β and IL-18 in H/R-exposed myocardial cells. Loss of miR-182-5p originated from exo-miR-182-5p inhibitor inverted the inhibitory effects of GSDMD knockdown on the levels of NLRP3 inflammasome-related cytokines. As shown in Fig. 6D–G, treatment with sh-GSDMD or exo-miR-182-5p mimic reduced LDH activity and ROS production while increasing myocardial cell viability and survival rate. However, treatment with exo-miR-182-5p inhibitor resulted in arrested cell viability and survival rate yet increased LDH activity and ROS production in the myocardial cells or GSDMD-deficient myocardial cells.

Furthermore, GSDMD knockdown or exo-miR-182-5p mimic co-culture decreased the protein levels of ASC, pro-caspase-1, and caspase-1 in the H/R-exposed myocardial cells. On the contrary, inhibition of exosomal miR-182-5p elevated the protein levels of those factors in H/R-exposed myocardial cells; however, exo-miR-182-5p-inhibitor counteracted the effects of GSDMD knockdown on those factors (Fig. 6H). The cleaved caspase-1/pro-caspase-1 ratio remained unaffected after any treatment.

miR-182-5p can prevent cardiac hypertrophy by regulating angiogenesis [32, 33] and overexpressed miR-182-5p weakens the tube formation potential of vascular endothelial cells [34]. Our results revealed a similar expression pattern of miR-182-5p in the umbilical vein endothelial cells (HUVECs) and myocardial cells (Supplementary Fig. 4A). Meanwhile, cell apoptosis in the myocardial tissues was assayed by TUNEL at 3, 12, and 24 h following intravenous injection of exosomes in the mice. Results revealed that cell apoptosis in the myocardial tissues was gradually reduced over time of exosome injection (Supplementary Fig. 4B). Altogether, miR-182-5p delivered by MSC-derived exosomes can downregulate GSDMD expression and thus suppresses H/R-induced pyroptosis and injury of myocardial cells.

### miR-182-5p delivered by MSC-derived exosomes inhibits I/R-induced myocardial cell pyroptosis and prevents I/R injury by downregulating GSDMD

**Fig. 7:**
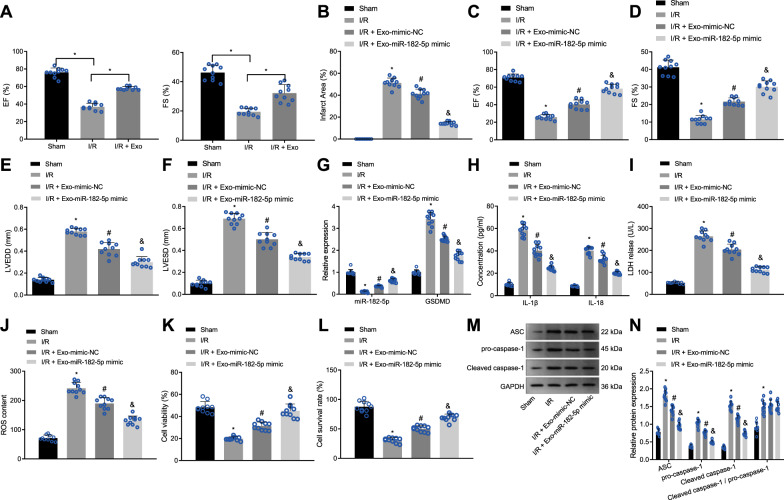
Exosomal miR-182-5p from MSCs diminishes I/R-induced myocardial cell pyroptosis and delays I/R injury in vivo. I/R-treated mice were injected with exo-miR-182-5p mimic (exosomes derived from miR-182-5p mimic-transfected MSCs) or exo-mimic-NC (exosomes derived from mimic-NC-transfected MSCs). **A** The echocardiography of cardiac functions of I/R mice; **B** MI size diagram calculated by the cross-sectional imaging; **C** Determination of LVEF of I/R mice; **D** Determination of LVFS of I/R mice; **E** Determination of LVESD of I/R mice; **F** Determination of LVEDD of I/R mice; **G** The expression of miR-182-5p and GSDMD mRNA level in myocardial tissues of I/R mice detected by RT-qPCR, normalized to U6 and GAPDH respectively; **H** The levels of IL-1β and IL-18 in myocardial tissue homogenate supernatant of I/R mice determined by ELISA; **I** LDH release in myocardial tissues of I/R mice detected by LDH kit; **J** ROS production in myocardial tissues of I/R mice measured by DCFH-DA; **K** The cell viability in myocardial tissues measured by Calcein-AM/PI double staining; **L** The cell survival rate in myocardial tissues of I/R mice measured by trypan blue staining; **M** Representative images of ASC, pro-caspase-1, and caspase-1 protein bands in myocardial tissues of I/R mice; **N** Western blot analysis of ASC, pro-caspase-1, and caspase-1 proteins in myocardial tissues of I/R mice, normalized to GAPDH. *N* = 10 for mice in each group. The comparison among multiple groups was conducted with one-way ANOVA, followed by Tukey’s post hoc test. Differences among groups at different time points were conducted by repeated-measures ANOVA with Bonferroni correction. *P < 0.05 vs. sham-operated mice. #*P* < 0.05 vs. I/R-treated mice. &*P* < 0.05 vs. I/R-treated mice injected with exo-mimic-NC.

To assess the impact of exosomal miR-182-5p delivery on myocardial I/R injury, the mice suffered myocardial I/R injury were given an intravenous injection of MSC-derived exosomes. Subsequently, we evaluated the cardiac function of mice by echocardiography, the results of which revealed alleviated cardiac dysfunction in mice after treatment with MSC-derived exosomes (Fig. 7A). In addition, MI size was found to be increased while the cardiac function was damaged in the I/R mice. Treatment with exo-mimic-NC led to reduced MI size and restored cardiac function while exo-miR-182-5p mimic induced a more obvious decline of MI size and recovery of cardiac function (Fig. 7B–F and Supplementary Fig. 5).

Next, we conducted RT-qPCR to document the miR-182-5p and GSDMD expression patterns in myocardial tissues. As seen in Fig. 7G, the expression of miR-182-5p was downregulated while that of GSDMD was upregulated in myocardial tissues of the I/R-rendered mice. exo-mimic-NC promoted miR-182-5p expression and decreased GSDMD expression, which was more evident following exo-miR-182-5p mimic. Furthermore, the levels of IL-1β and IL-18 in the tissue homogenate supernatant, LDH activity, ROS production, and the protein levels of ASC, pro-caspase-1, and caspase-1 were all decreased while cell viability and survival rate were enhanced in the myocardial tissues of I/R-treated mice injected with exo-mimic-NC. These changes were more prominent upon treatment with exo-miR-182-5p mimic (Fig. 7H–N). RNA-fluorescence in situ hybridization (FISH) assay showed downregulation of miR-182-5p both in mouse myocardial tissues and epidermal tissues following I/R modeling. exo-mimic-NC increased miR-182-5p expression in the I/R-operated mice, which was more evident upon exo-miR-182-5p mimic injection (Supplementary Fig. 6). These results showed that miR-182-5p delivered by MSC-derived exosomes could inhibit I/R-induced myocardial cell pyroptosis and arrest I/R injury by downregulating GSDMD in vivo.

## Discussion

MSC-derived exosomes possess the ability to restore bioenergetics, decrease oxidative stress, and enhance myocardial cell viability, and can thereby promote cardiac function after the occurrence of myocardial I/R injury [35]. Owing to the fact that MSC-derived exosomes can further control stem cell differentiation by carrying and transferring miRNAs, stem cell-based exosome therapeutic regimens have been highlighted as effective means for cardiac regeneration after ischemic heart disease [36]. In the current study, we set out to elucidate the possible therapeutic effects of exosomal miR-182-5p on myocardial I/R damage with the help of in vitro H/R and in vivo I/R models. Our composite findings identified that miR-182-5p shuttled by MSC-originated exosomes could ameliorate myocardial I/R injury by reducing the GSDMD expression.

Firstly, findings attained in the current study demonstrated that miR-182-5p was poorly-expressed in myocardial tissues from I/R mice. This particular finding is in line with a previous study, which documented downregulated levels of miR-182 in the pineal gland post-hypoxia-ischemia brain injury [37]. Similar expression patterns have also been previously uncovered for miR-199a-3p and miR-214, which were decreased in H9c2 cells in response to I/R. More importantly, both the latter miRNAs are known to regulate myocardial cell survival to exert their cardioprotective effects [20, 38]. Meanwhile, poorly-expressed miR-182-5p levels were also previously observed in H9c2 myocardial cells after hypoxia-induced injury [20]. In addition, the protective effects of miR-182 against I/R injury and myocardial cell death have been reported [21]. Together, our data in conjunction with existing data illustrate that miR-182-5p confers cardioprotective effects against myocardial I/R injury.

Furthermore, our findings substantiated that miR-182-5p could be carried and transferred by exosomes derived by MSCs. Inherently, exosomes are known to function as vesicles to transfer functional miRNAs from host cells to transplanted cells [39, 40]. Moreover, reports also suggest that MSC-derived exosomes can exert a cardioprotective effect in myocardial I/R injury [40]. For instance, miR-182 loaded in exosomes derived from MSCs was previously demonstrated to impart a cardioprotective effect against myocardial I/R injury [41]. In addition, enhancement of miR-182-5p induced by ganoderic acid A could antagonize hypoxia-induced myocardial cell injury [20]. The above findings are partly concordant with our present finding that restoration of miR-182-5p via exosome transfer in vitro and in vivo diminished I/R-induced myocardial cell injury. What’s noteworthy is that I/R injury accounts for up to 50% of the final MI size [42]. On the other hand, the administration of purified exosomes was proven to reduce MI size in a murine I/R model [40]. Consistently, these data indicate that miR-182-5p delivered by exosomes reduced MI size and improved mouse cardiac function.

Additionally, we further encountered that exosomal miR-182-5p transfer decreased NLRP3 inflammasome activation, cell oxidative stress, and pyroptosis, leading to alleviated myocardial I/R injury. NLRP3 is regarded as a general sensor of cell stress, while another miRNA (miR-223) was recently reported to reduce NLRP3 inflammasome activity by binding to NLRP3 3’UTR to repress its expression [43]. The process of pyroptosis is known to be triggered by the NLRP3 protein, through the activation of pyroptosis-related proteins, which include ASC and caspase-1. Meanwhile, caspase-1 is known to induce the secretion of IL-1β and IL-18 by bridging NLRP3 protein with pro-caspase-1 within the inflammasome complex [44, 45]. On the other hand, overexpression of miR-182-5p downregulates Toll-like receptor 4 (TLR4) to inactivate pro-inflammatory cytokines tumor necrosis factor α and IL-6, thereby ameliorating liver I/R injury [45]. Furthermore, extensive efforts made by our peers have also identified oxidative stress induced by reperfusion as the primary contributor to I/R injury [46]. Meanwhile, restoration of miR-182-5p was also reported to reduce atherosclerosis-induced oxidative stress and apoptosis by means of downregulating TLR4 and restraining ROS production [47]. Our current results expanded on this and highlighted that miR-182-5p reduced the expression of ASC, pro-caspase-1, and that caspase-1 might be an underlying factor related to a miR-182-5p-mediated reduction of pyroptosis meriting further investigation.

Furthermore, miR-182-5p targeted and reduced the GSDMD expression in the I/R myocardium and diminished GSDMD-mediated apoptosis in H/R-exposed myocardial cells. These data concur with a previous study [48], wherein GSDMD was indicated as an essential component for the regulation of NLRP3 inflammasome-evoked release of IL-1β and IL-18 secretion [48]. Meanwhile, inflammatory caspase-induced cleavage of GSDMD allows its N-terminal domain to connect with membrane lipids and form pores, which is known to elicit pyroptotic cell death [49]. The pore-forming GSDMD protein is also necessary for IL-1β secretion from living macrophages following exposure to inflammasome activators [50]. When GSDMD is reduced, cells can resist pyroptosis that would otherwise be provoked via cytosolic lipopolysaccharide or ligands of canonical inflammasome [51]. In addition, it has been demonstrated that inhibition of hypoxia-induced inflammasome activation is accompanied by reduced expression levels of NLRP3, caspase-1, p20, and GSDMD N-terminal fragment while the suppression on pyroptosis is evidenced by diminished LDH release in H9c2 cells [52]. The aforementioned data is highly-consistent with our findings that GSDMD knockdown could reduce H/R-induced myocardial cell apoptosis and pyroptosis. As shown by our data, when the generation of GSDMD N from GSDMD induced by activated caspase was blocked by VX-765, the production of IL-1β and IL-18 in the H/R-exposed myocardial cells exhibited a reduction. Together, these findings and data indicate that reduced expressions of GSDMD might be the underlying mechanism for reduced inflammation and improved survival in I/R-exposed myocardial cells mediated by exosomal miR-182-5p.

Overall, our investigation highlighted the protective role of exosomal miR-182-5p in myocardial cells during I/R injury through decreasing myocardial cell pyroptosis via modulating the miR-182-5p/GSDMD axis. Multiple studies have further reported miR-182-5p as a potential target against anoxic damage or apoptosis of myocardial cells during I/R injury. For instance, miR-182-5p inhibition was indicated to protect myocardial cells from hypoxia-induced apoptosis by targeting CIAPIN1 [53]. Likewise, miR-182-5p can further alleviate liver I/R injury by downregulating TLR4 [45]. In addition, another research has proposed that the miR-182-5p content in the blood can be indicative of unprotected left main coronary artery disease [54]. All these findings validated the protective role of miR-182-5p. Our experimental data demonstrated that miR-182-5p downregulated GSDMD to suppress pyroptosis. Based on previous reports, it was inferred that miR-182-5p may mediate other cell apoptosis-related signaling pathways including myocardial cell apoptosis in I/R, which will be an important research topic in our future studies.

**Fig. 8:**
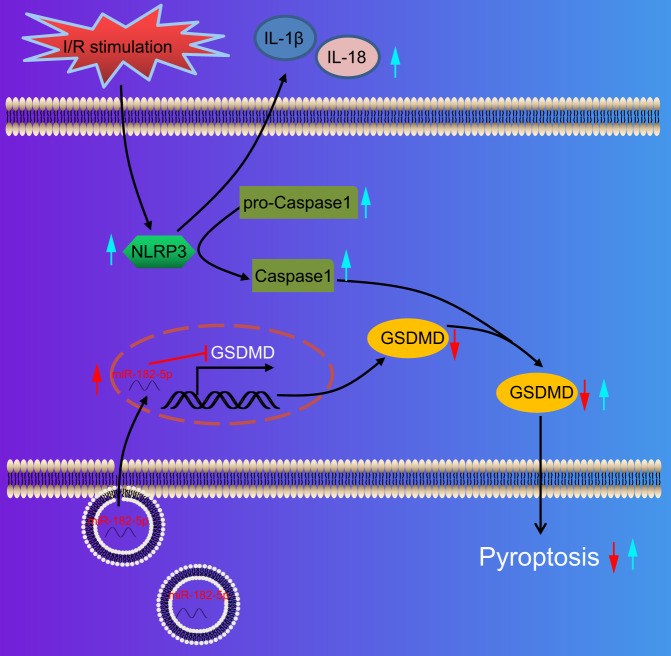
A schematic diagram showing potential molecular mechanisms of exosomal miR-182-5p targeting GSDMD in myocardial I/R injury. miR-182-5p shuttled by exosomes from MSCs can be internalized by myocardial cells, and then targets GSDMD and reduces its expression, suppressing myocardial cell injury and pyroptosis, and delaying the myocardial I/R injury in mice.

Altogether, findings obtained in the current study indicate that miR-182-5p delivered by MSC-derived exosomes may protect against myocardial cells from I/R-evoked inflammation, apoptosis, and injury (for details please refer to Fig. 8). These effects may be attributed to miR-182-5p-mediated inhibition of GSDMD. Additionally, the exosomal miR-182-5p could enhance the rescue of cardiac function and inhibit MI in mice with myocardial I/R injury, therefore, exhibiting promise as appealing therapeutic targets for future MI treatments. It is also important to note that the efficacy of exosomal miRNA transfer may differ based on the type of technologies used, which may bring certain clinical challenges in translational studies. In addition, future large-scale mouse studies are warranted to illustrate the mechanism by which exosomal miR-182-5p protects against myocardial I/R injury.

## Materials and methods

### Ethics statement

Animal experiments were conducted in compliance with the Guide for the Care and Use of Laboratory Animal (8^th^ Edition, National Institute of Health, 2011) and with the approval of the Animal Ethics Committee of North Sichuan Medical College. Extensive measures were taken to minimize animal suffering in the study.

### Myocardial I/R mouse model establishment

Male healthy C57BL/6 wild type mice (aged: 7–11; weighing 18 g; Shanghai SJA Laboratory Animal Co., Ltd., Shanghai, China) were individually caged in the SPF laboratory of 60–65% humidity, at 22–25 °C, under a 12-h light/dark cycle, with ad libitum access to food and water, for 1 week before the experiment. The health status of the mice was monitored prior to the experimentation. The mouse model of I**/**R was developed by a 30-min occlusion procedure of the LAD and reperfusion based on existing protocols [55]. Briefly, the mice (*n* = 30) were anesthetized by intraperitoneal injection with 3% pentobarbital sodium (Takara Bio Inc., Otsu, Shiga, Japan) in the induction room, and then supported using oxygen via a small-animal ventilator. A horizontal incision was then made between the fourth and fifth ribs of the left chest to expose the heart and was subsequently sutured with a 6-0 silk knot at the proximal LAD (2–3 mm). The ligation area was white with evident arrhythmia, thus indicative of successful MI injury. The knot was untied 30 min post-MI. Successful reperfusion was validated by epicardial congestion. Another group of ten mice were sham-operated without ligation of LAD as control. In addition, the I/R-rendered mice were further injected with 10 μg of Exo-mimic-NC or 10 μg of exo-miR-182-5p mimic which was dissolved in 10 μL phosphate buffer saline (PBS) prior to use, at the front and outside of the visible injury area [56].

### Measurement of cardiac function

Three hours after reperfusion, the mouse cardiac function was assessed via echocardiographic and hemodynamic measurements by left ventricular catheterization. Briefly, mice (*n* = 3) in each group were intraperitoneally given 50 mg/kg pentobarbital sodium (57-33-0; Shanghai Beizhuo Biotechnology Co., Ltd., Shanghai, China) for anesthesia, intubated, and then mechanically ventilated with oxygen. A catheter was inserted in the left ventricle through the mitral valve for connection with a pressure transducer (AD Instruments, New South Wales, Australia). Data were obtained by a MacLab/4 s data acquisition system (AD Instruments) and a Power Macintosh 7200/90 computer (Apple Inc., Cupertino, CA). End-diastolic pressure in the left ventricle was adjusted to 4–6 mmHg prior to the real-time data recording. Left ventricular ejection fraction (LVEF), left ventricular fractional shortening (LVFS), left ventricular end-systolic diameter (LVESD), and left ventricular end-diastolic diameter (LVEDD) were measured. The relative data are listed in Supplementary Table 1.

### Evans blue and 2,3,5-triphenyltetrazolium chloride (TTC) double staining

After 24 h of reperfusion, the mice were anesthetized and the LAD was re-occluded. Evans blue dye (2%) was delivered to the heart via the carotid artery for identification of the ischemic regions. The non-ischemic myocardium was stained blue while the ischemic regions remained unstained. The mice were then euthanized using an intravenous injection of 10% KCl solution. The heart was cautiously harvested and the left ventricle was sliced into 3–4 mm-thick sections. Freshly prepared 5% TTC was utilized to incubate with the sections in dark for 15 min at 37 °C to distinguish the infarcted regions and non-infarcted regions (infarcted regions were not stained, and non-infarcted regions were stained red). Each sample was documented with a digital camera through which the MI size was quantified using the Sigma Scan Pro 4.0 software (Aspire Software International, Ashburn, VA) as described previously [57].

### Establishment of the H/R-induced myocardial cell models

Myocardial cells were isolated utilizing the methods as stated in the previous literature [58]. Briefly, the left ventricular tissues, obtained from C57BL/6 suckling mouse pups (aged 1–3 days; Shanghai SJA Laboratory Animal Co., Ltd) were incised into 1 mm^3^ blocks. Post detachment using 0.1% trypsin, the cells were filtered through a 40 μm cell strainer. After centrifugation, the myocardial cells were isolated and suspended in high-glucose Dulbecco’s modified Eagle’s medium (DMEM, Gibco BRL, Life Technologies Inc., Grand Island, NY) replenished with 20% fetal bovine serum (FBS) and 0.1 μM 5-Bromo-2-deoxyuridine (Gibco). Subsequently, the myocardial cells were purified using the differential adherence method, stained with trypan blue, and counted. Myocardial cells (5 × 10^5^ cells/mL) were then seeded into a 24-well plate and subjected to incubation at 37 °C with an incubator (Thermo Fisher Scientific, Waltham, MA) that contained 5% CO_2_ and saturated humidity.

The purity of myocardial cells was assessed by immunofluorescence detection of cTnI. The culture medium was removed after 3 days of culture, and then myocardial cells were fixed using 4% paraformaldehyde for 10–20 min. The cells were probed employing the polyclonal antibody to cTnI diluted at 1:10 (ab209809, Abcam, Cambridge, UK) and subsequently re-probed with the fluorescein isothiocyanate (FITC)-coupled secondary antibody. After sealing, the myocardial cells were observed under a fluorescence microscope. Six fields were screened at randomization and photographed to count the fluorescence-positive cells as myocardial cells (n1). The total number of cells was counted as N1. The ratio of myocardial cells was expressed as n1/N1 × 100%.

Myocardial cells were then exposed to hypoxic conditions for 24 h in a hypoxic incubator containing a combination of 1% O_2_, 5% CO_2_, and 94% N_2_ followed by reoxygenation for 12 h in an incubator with a combination of 21% O_2_, 5% CO_2_, and 74% N_2_.

### Trypan blue staining

Mouse tissue samples were taken, washed, and cut into 1 mm^3^ tissue pieces, which were then digested with 0.1% trypsin, filtered with a 40 μm pore size filter, and centrifuged. The precipitated cells were resuspended in a medium without phenol red and serum. Next, 5% trypan blue solution prepared using 5 g of trypan blue (T6146-5G, Sigma-Aldrich Chemical Company, St Louis, MO) added with sterile PBS were diluted to ten times with cell culture medium, and mixed with the cells. Lastly, the cells were counted in the captured microscopic images under an inverted microscope within 3 min.

### HUVEC culture

The culture of HUVECs supplied by American Type Culture Collection (ATCC; Manassas, VA) was proceeded in Roswell Park Memorial Institute 1640 medium (Thermo Fisher Scientific) containing 20% FBS, 60 μg/mL of endothelial cell growth supplement (BD Biosciences, San Jose, CA), 100 U/mL of penicillin, and 100 μg/mL of streptomycin (Thermo Fisher Scientific) at 37 °C under saturated humidity with 5% CO_2_.

### Immunohistochemistry staining

Paraffin-embedded mouse myocardium slices were dewaxed and antigen retrieved by the standardized procedures. Slices were treated with 3% H_2_O_2_ in methanol for 20 min to deactivate the endogenous peroxidase. After a 3-min rinse with 0.1 M PBS, the slices were blocked employing normal goat serum (C-0005) sourced from Haoran Bio Technologies Co., Ltd. (Shanghai, China). The rabbit anti-human GSDMD (1: 1000, ab219800, Abcam) served as the primary antibody for overnight incubation at 4 °C. On the following day, re-probing was completed with goat anti-rabbit immunoglobulin G (IgG) (ab6785, 1: 1000, Abcam) for 20 min at 37 °C. Next, incubation at 37 °C with horseradish peroxidase (HRP)-coupled streptavidin solution (0343-10000U, Imunbio Biotechnology Co., Ltd., Beijing, China) lasted for 20 min. The slices were then stained with diaminobenzidine (ST033, Guangzhou Whiga Technology Co., Ltd., Guangdong, China) and counter-dyed utilizing hematoxylin (PT001, Shanghai Bogoo Biological Technology Co., Ltd., Shanghai, China). After immersion in 1% ammonia, the slices were processed with conventional procedures. Finally, the dyed slices were documented utilizing microscopic images.

### RNA isolation and quantification

Total RNA content was harvested from the tissues and cells as per the specifications of the TRIzol reagent (Invitrogen, Carlsbad, CA). For mRNA detection, after determination of the RNA concentration, the complementary DNA (cDNA) was generated after RT of RNA content (1 µg) by the Primescript^TM^ RT Reagent Kit with gDNA Eraser (RR037A, Takara). For miRNA detection, PolyA Tailing Reverse Transcription Kit (B532451, Sangon Biotech Co., Ltd., Shanghai, China; containing universal PCR reverse primer for miRNA and U6) was adopted for RT, with PolyA-containing cDNA obtained. The polymerase chain reaction (PCR) was performed by an SYBR^®^ Premix Ex Taq^TM^ (Tli RNase H Plus) reagent kit (RR820A, Takara) and an ABI7500 real-time qPCR system (Thermo Fisher Scientific). miR-182-5p was quantitated by real-time qPCR using a TaqMan miRNA Assays Kit (Thermo Fisher Scientific). Primer sequences from GenePharma (Shanghai, China) are presented in Supplementary Table 2. Glyceraldehyde-3-phosphate dehydrogenase (GAPDH) and U6 were selected as endogenous controls for the GSDMD protein and miR-182-5p respectively. Relative quantification of the target genes was performed based on the 2^−ΔΔCt^ method [59].

### Western blot analysis

Total protein was extracted from the tissues and cells by RIPA lysis buffer with a 1% protease inhibitor. After centrifugation, the supernatant was collected. A bicinchoninic acid (BCA) kit (23227, Thermo Fisher Scientific) was adopted for the protein concentration determination. Protein separation was conducted by polyacrylamide gel electrophoresis and then loaded onto the polyvinylidene fluoride membrane. After blockade by 5% skimmed milk, an incubation was conducted with the use of corresponding primary antibodies against apoptosis-associated speck-like protein containing a C-terminal caspase recruitment domain (ASC) (1: 100, ab18193, Abcam), pro-caspase-1 (1: 1000, ab16883, Abcam), caspase-1 (1: 100, AF4005, Affinity Biosciences, CN), GSDMD (1: 1000, ab209845, Abcam), NLRP3 (1: 500, ab214185, Abcam), interleukin-1β (IL-1β; 1: 100, ab71495, Abcam), CD63 (1: 1000, ab216130, Abcam), heat-shock protein-70 (HSP70) (1: 000, ab2787, Abcam), tumor susceptibility gene 101 (TSG101) (1: 1000, ab125011, Abcam), Alix (1: 1000, ab186429, Abcam), Calnexin (1: 100, ab22595, Abcam), and GAPDH (1: 5000, ab8245, Abcam) overnight at 4 °C. HRP-tagged goat anti-rabbit IgG (1: 20000, ab205718, Abcam) was employed for 90 min of incubation. Subsequently, enhanced chemiluminescence (NCI4106, Pierce, Rockford, IL) was adopted to visualize protein bands. The gray intensity of each band was measured by the ImageJ 1.48U software (Bio-Rad, Hercules, CA). Data were presented as the relative ratio of the gray intensity between the target protein and GAPDH (loading control).

### Measurements of LDH release and ROS production

In compliance with the instructions of the LDH assay kit available from Nanjing Jiancheng Bioengineering Institute (Nanjing, China), the tissue block (0.1–0.2 g) was rinsed in ice-cold normal saline to remove the blood, dried with filter paper, loaded into a homogenization tube. Thereafter, the tube was added with homogenization medium (0.86% normal saline) at the ratio of weight (g): volume (mL) =1:9 for homogenization, and the tissues were chopped with small ophthalmic scissors in an ice water bath. The homogenate was produced in a homogenizer. For cells, they were centrifuged and the cell pellet was collected. The cell suspension was centrifuged at 1000 r/min for 10 min, with the supernatant discarded and the cell pellet harvested. The cell pellet was washed and prepared into a homogenate. The absorbance value at 450 nm was measured using a microplate reader.

The measurement of ROS was then conducted. Cells after transfection and myocardial tissues were collected. Myocardial tissues were fixed in 10% formalin, frozen in liquid nitrogen, and preserved at −80 °C. Next, the tissues were loaded on the tissue support with optimum cutting temperature compound-embedded and prepared into 10-μm-thick sections, which were then loaded onto the slides. Afterward, the sections were added with 10 umol/L of reactive oxygen fluorescent probe dihydroethidium diluted by PBS (pH 7.4) for 30 min of incubation at 37 °C, followed by washing with PBS. The cell red emission images through the N21 filter were captured under the fluorescence microscope. For cells, they were digested, washed, and loaded with 15 μM DCFH-DA to intracellularly convert DCFH for 15 min in the dark. The cells were subsequently scraped off in 1 mL of ice-cold PBS whereupon 2 × 10^6^ cells were incubated. LPS-220B spectrofluorometer (Photon Technology International, Bermingham, NJ) was utilized for recording the fluorescence excited at 485 nm and emitted at 535 nm for 20 min. The difference between the end points and the start points was compared for the calculation of the DCF fluorescence units.

### Cell viability and apoptosis measurement

Mouse tissue samples were taken, washed, and cut into 1 mm^3^ tissue pieces, which were then digested with 0.1% trypsin, filtered with a 40 μm pore size filter, and centrifuged to collect the precipitated cells. The cells were resuspended in a medium without phenol red and serum. Calcein-AM/PI Cell Viability/Cytotoxicity Assay Kit (C2015M, Beyotime) was applied for measuring the cell viability rate. Cells were mixed with 1× Assay Buffer, and dyed by 2 μM Calcein-AM and 4.5 μM PI at 37 °C for 30 min. A fluorescence microscope (Olympus IX51) was utilized for observing and Image Pro advanced software for evaluating the average fluorescence intensity [29].

The tissues were prepared into 4-μm-thick sections before experimentation. The apoptosis in cells and tissues was detected using TUNEL Fluorometric Kit (Promega, Madison, WI), with DAPI (D8200, Solarbio, Beijing, China) utilized for nuclear staining. Images were acquired with a fluorescence microscope at a magnification of ×400, and positive cells were counted at a magnification of ×200, with at least ten fields of view checked for each sample [29].

### ELISA

IL-1β and IL-18 levels in the supernatant of cells and tissue homogenate were measured by IL-1β (ab100704, Abcam) and IL-18 (ab216165, Abcam) ELISA kits following the provided instructions. The optical density (OD) value at 450 nm was recorded using a Synergy 2 microplate.

### Isolation, culture, and identification of MSCs

Well-grown C57BL/6 mice were euthanized and soaked in alcohol for 10 min. The femurs and tibias of the mice were removed in a sterile environment, with the meat discarded using the instrument, and then washed in a plate containing DMEM (Gibco). Both ends of the femur and tibia were removed with clean and sterile scissors, after which the bone marrow cells were delivered using a DMEM-contained syringe into a 15 mL centrifuge tube for 3-min centrifugation at 1500 r/min, followed by removal of the supernatant. Cells were resuspended in a 5% CO_2_ incubator at 37 °C employing DMEM replenished with 10% FBS (Gibco) and 100 U/mL penicillin-streptomycin (Gibco). After three days, the medium was renewed to get rid of non-adherent cells, and meanwhile, the changes of cell morphology were visualized, photographed, and recorded. The cells of 80–90% confluence were subcultured and those at passage 3 were collected for later use.

MSCs at the 80% confluence were collected, fixed overnight in 4% paraformaldehyde at 4 °C. The cells were directly labeled for 1 h with FITC-coupled antibodies to CD34, CD11b, CD45, CD90, CD105, and CD73 (all sourced from BD Biosciences Pharmingen, San Jose, CA), and then with FITC-coupled goat anti-mouse IgG (1:200; BD Biosciences) for 1 h. A sum of 1 × 10^4^ FITC-marked cells were counted with BD FACS Calibur [60].

In addition, MSCs at passage 3–5 were cultured in OriCell osteogenic, adipogenic, or chondrogenic differentiation medium (Cyagen, Guangzhou, China). Alizarin Red S, Oil Red O, and Alcian Blue staining tests were proceeded for the identification of MSCs by assessing the osteogenic, adipogenic, and chondrogenic differentiation properties [61].

### Extraction and characterization of MSC-derived exosomes

MSCs at passage 3 (1 × 10^7^ cells, 1 × 10^6^ cells/mL, 10 mL) were incubated overnight with serum-free DMEM in a petri dish (90 mm). Upon attaining 80–90% confluence, the MSCs were incubated for 24 h, followed by the collection of the supernatant. The supernatant was harvested after sequential centrifugations (at 350×*g*, 2000×*g*, and at 12,000×*g*) for 10 min at 4 °C. The attained supernatant was filtered on a 0.22-μm filter to eliminate any large particles. The pellet was obtained through centrifugation at 120,000×*g* for 70 min at 4 °C, resuspended in PBS, and purified by repeated centrifugation at 120,000×*g* for 70 min at 4 °C. The purified pellet (exosomes) was suspended in PBS, and preserved at −80 °C for subsequent experimentation with the exosome output shown in Supplementary Fig. 7 [62, 63].

The exosome surface marker CD63 (ab217345, Abcam) was assayed using flow cytometry. Exosomes were resuspended employing 1 mL PBS containing 1% bovine serum albumin (BSA) at ambient temperature for 30 min. Five minutes post centrifugation, the supernatant was discarded and exosomes were resuspended in EP tubes with 200 µL PBS and subsequently allowed to incubate with the phycoerythrin (PE)-conjugated anti-CD63 antibody, isotype control (PE-conjugated anti-human IgG), or blank control (no-antibody) for 30 min. After that, another 5-min of centrifugation was conducted, followed by resuspension in PBS containing 1% BSA.

TEM observation: the mixture (5 μL) of exosome suspension with 4% paraformaldehyde was added to the carbon-coated grid, and then stained with 1% phosphotungstic acid. Images were acquired using a TEM (HT7830, Hitachi, Tokyo, Japan).

NTA procedures: the collected exosomes were diluted using PBS to 10^6^/mL−10^9^/mL and loaded into the Nanosight NS300 analyzer (Malvern, UK) using a 1 mL syringe for analysis.

Western blot analysis of exosome markers: exosome particles were immersed in RIPA buffer, in which which the protein was quantified utilizing BCA kit (A53226, Thermo Fisher Scientific). The expression of CD63, HSP70, TSG 101, Alix, and Calnexin in exosomes was detected using Western blot analysis.

### Dual-luciferase reporter assay

GSDMD 3′UTR sequences carrying putative miR-182-5p binding sites and its mutant sites were independently inserted into a pmirGLO luciferase reporter vector, regarded as the GSDMD-WT or GSDMD-MUT plasmid. The miR-182-5p mimic and mimic-NC were co-transduced with the constructed GSDMD-WT or GSDMD-MUT into the human embryonic kidney (HEK)-293T cells (ATCC; cultured in DMEM containing 10% FBS and 1% penicillin-streptomycin in a 5% CO_2_, 37 °C incubator). Twenty-four hours post transduction, the cells were lysed. The lysate was centrifuged at 12,000 rpm for 1 min and the supernatant attained here was preserved for use. A Dual-Luciferase^®^ Reporter Assay System (E1910, Promega) was adopted for luciferase activity measurement. Relative luciferase activity was presented as the ratio of Firefly to the internal reference Renilla luciferase.

### Cell transfection

H/R-exposed myocardial cells in the logarithmic growth period were seeded into a six-well plate (4 × 10^5^ cells/well). The myocardial cells of 80–90% confluence were manipulated with the overexpression plasmids, short hairpin RNAs (shRNAs), mimics, and inhibitors using Lipofectamine 2000 reagent (11668-019, Invitrogen) in strict compliance with the manufacturer’s instructions. The plasmids (sh-GSDMD, miR-182-5p mimic, miR-182-5p inhibitor, corresponding sh-NC, mimic-NC, and inhibitor-NC) were supplied by GenePharma (Shanghai, China).

MSCs were cultured at a density of 2 × 10^5^ cells/well with the DMEM without penicillin-streptomycin for 24 h. Upon attaining 70–90% confluence, the MSCs were transfected with mimic-NC/inhibitor-NC or miR-182-5p mimic/inhibitor using the TransIT^®^-2020 transfection reagent (MIR5404, Mirus, Madison, WI). The medium was replaced 6 h following transfection. After 48 h, the MSCs were harvested and used for follow-up experimentation.

### Co-culture of MSCs or exosomes with myocardial cells

MSCs were allowed to grow to a confluence of 80–90% after seeding into a six-well plate (1 × 10^6^ cells/well). Then, the MSCs were then treated with 10% GW4869 (an inhibitor of exosome release) (D1692-5MG, Sigma-Aldrich) or 0.005% dimethyl sulfoxide (DMSO) (as the control). Twenty-four hours post-incubation, the MSCs and supernatants were preserved for further use.

MSCs (1 × 10^4^ cells/well) treated with DMSO or GW4869 were cultured in the basolateral compartment of a 24-well transwell system (0.4 μm pore size). The apical compartment contained myocardial cells exposed to H/R and without treatment or transfected with the mimics or inhibitors. Twenty-four hours later, the myocardial cells were attained for miR-182-5p and GSDMD expression pattern characterization. Exosomes were stained with the red lipophilic fluorescent dye PKH26 (MINI67-1KT, Sigma-Aldrich). After staining, the PKH26-tagged exosomes were co-cultured with the myocardial cells at 50–60% confluence seeded into the 24-well plate for 48 h. H/R-exposed myocardial cells were manipulated with sh-NC, sh-GSDMD, or co-incubated with Exo-mimic-NC, exo-miR-182-5p mimic, Exo-inhibitor-NC, or exo-miR-182-5p inhibitor, or co-incubated with exo-miR-182-5p inhibitor and manipulated with sh-GSDMD in combination. Myocardial cells were then harvested and monitored under observation with an inverted fluorescence microscope.

### RNA-FISH assay

The myocardial tissues or epidermal tissues of mice embedded in paraffin were sliced into sections, and heated at 65 °C for 4 h and at 73 °C for 2 min. After rehydration using xylene, ethanol, and distilled water, respectively, the sections were treated with SSC for 5 min, then with proteinase K for 30 min and with SSC three times (5 min/time) and finally fixed with formaldehyde for 10 min. Following treatment using ethanol, 1.5 μL of miR-182-5p probes designed by GenePharma (F22101/50; Shanghai, China) were applied for hybridization and a fluorescence microscope was employed for observation.

### Statistical analysis

Data processing and analyses were implemented with the application of the SPSS 21.0 statistical software (IBM Corp., Armonk, NY). Measurement data were summarized as mean ± standard deviation. Comparison between two groups was performed using the unpaired *t*-test. Multigroup comparison was proceeded employing one-way analysis of variance (ANOVA), followed by fTukey’s post hoc test. Differences among multiple groups concerning time-based data were assessed with repeated-measures ANOVA. Pearson’s correlation analysis was utilized to verify the relevance between miR-182-5p and GSDMD expression in the I/R mouse models. Differences were deemed to be significant with the value of *p* < 0.05.

## Supplementary information

Supplementary Figures

Supplementary Tables

Original Western Blots

## Data Availability

The data that support the findings of this study are available from the corresponding author upon reasonable request.
