# Supplementary material for: Mesenchymal stem cell-derived exosomal microRNA-182-5p alleviates myocardial ischemia/reperfusion injury by targeting GSDMD in mice
Source: Cell Death Discov. 2022 Apr 14;8:202. doi: 10.1038/s41420-022-00909-6 (PMC9010441; doi:10.1038/s41420-022-00909-6)
Supplement: Supplementary file 1 — Supplementary Figures [file 41420_2022_909_MOESM1_ESM.docx]

**
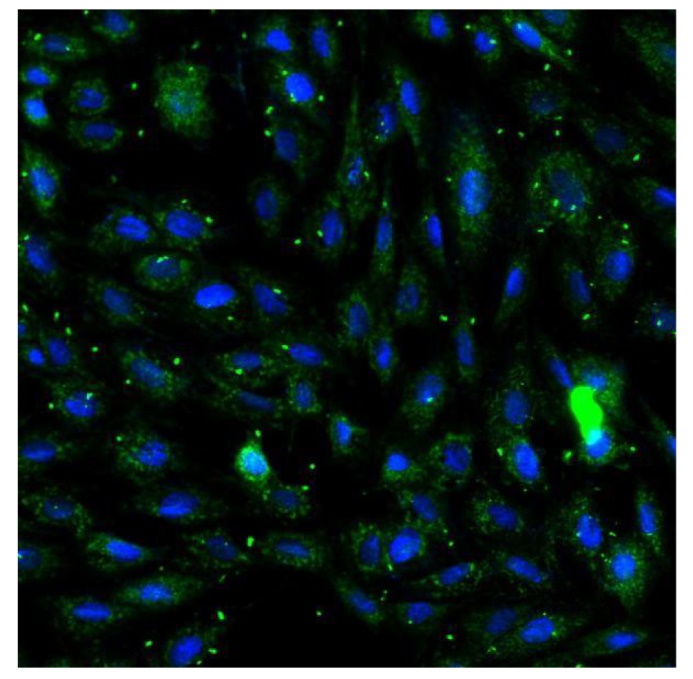
**

**Supplementary Figure 1** Immunofluorescence detection of cTnI protein in the isolated myocardial cells (× 200).

**
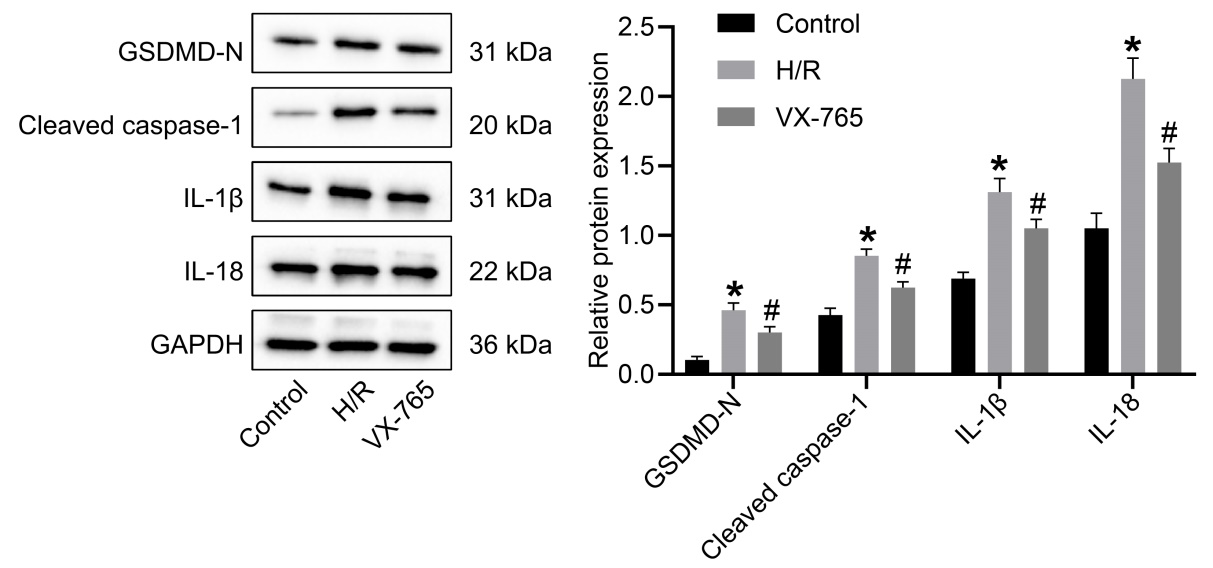
**

**Supplementary Figure 2** VX-765 treatment can reduce the levels of cleaved caspase-1, GSDMD-N, IL-1β, and IL-18 in H/R-exposed myocardial cells. Western blot analysis of GSDMD-N, cleavced-caspase-1, IL-1β, and IL-18 proteins in untreated myocardial cells or H/R-exposed myocardial cells after treatment with VX-765, normalized to GAPDH. * *p* < 0.05 *vs.* untreated myocardial cells, # *p* < 0.05 *vs.* H/R-exposed myocardial cells. Each cell experiment was repeated three times independently.

**
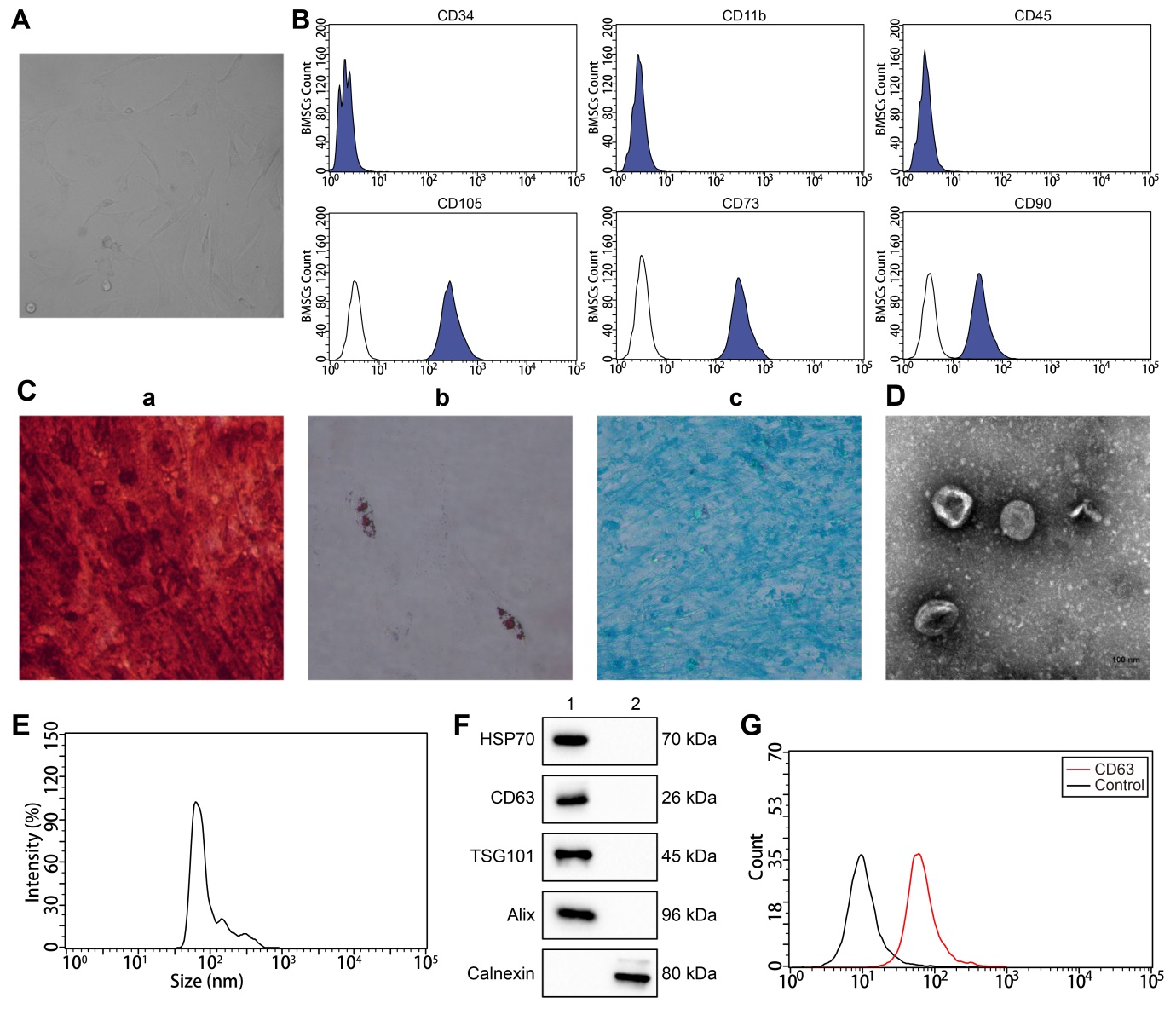
**

**Supplementary Figure 3** MSCs and MSC-derived exosomes are successfully isolated. A, Morphological characteristics of MSCs observed under an inverted microscope (× 100); B, Expression of MSC surface markers assessed by flow cytometry; C-a, The osteogenic differentiation potential of MSCs (× 200); C-b, The adipogenic differentiation potential of MSCs (× 200); C-c, The chondrogenic differentiation potential of MSCs (× 400); D, Morphological characteristics of MSC-derived exosomes observed under a TEM; E, The exosome diameter analyzed by NTA; F, Western blot analysis of CD63, HSP70, TSG101, and Alix proteins in MSC-derived exosomes (1) and the supernatants (2); G, The expression of exosome surface marker CD63 assessed by flow cytometry. Measurement data were expressed as mean ± standard deviation. The comparison between two groups was conducted by unpaired *t*-test. Each experiment was repeated three times independently.

**
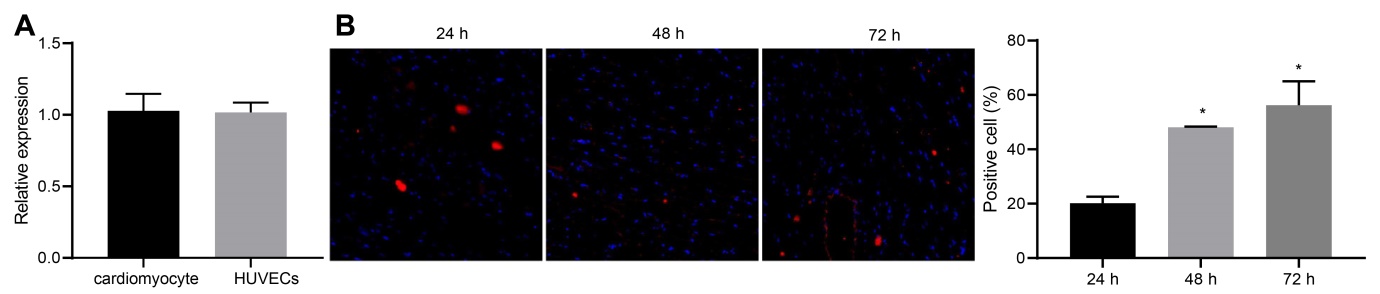
**

**Supplementary Figure 4** miR-182-5p loaded in MSC-derived exosomes suppresses myocardial cell apoptosis. A, The relative expression of miR-182-5p in mouse myocardial cells and HUVECs determined by RT-qPCR; B, TUNEL-positive cells in myocardial tissues of mice. Red indicates TUNEL-positive cells while blue indicates DAPI staining. Each experiment was repeated three times independently.

**
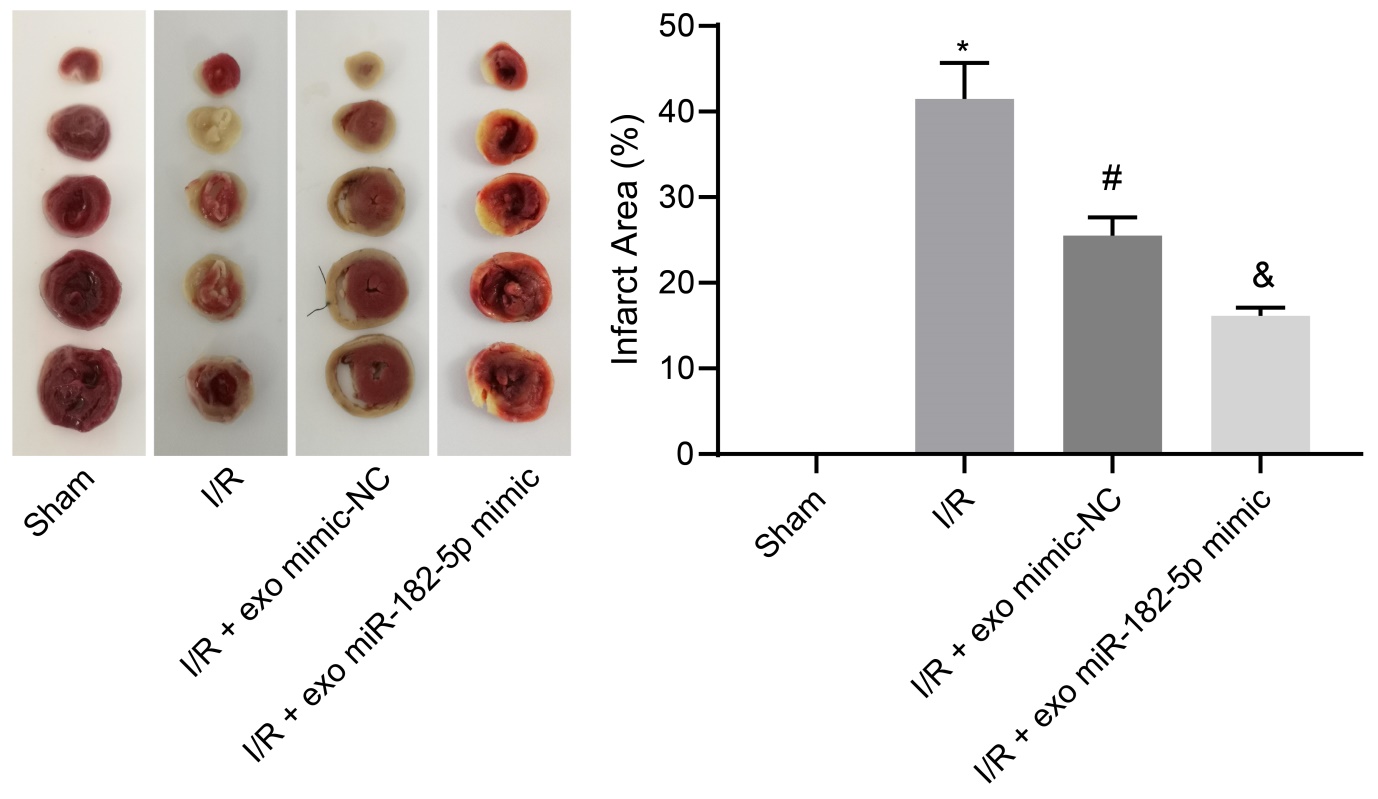
**

**Supplementary Figure 5** miR-182-5p loaded in MSC-derived exosomes reduces MI size. Infarct area was determined by TTC/Evans blue staining. * *p* < 0.05 *vs.* sham-operated mice, # *p* < 0.05 *vs.* I/R mice, & *p* < 0.05 *vs.* I/R mice treated with Exo mimic-NC. N = 10 for mice in each group.

**
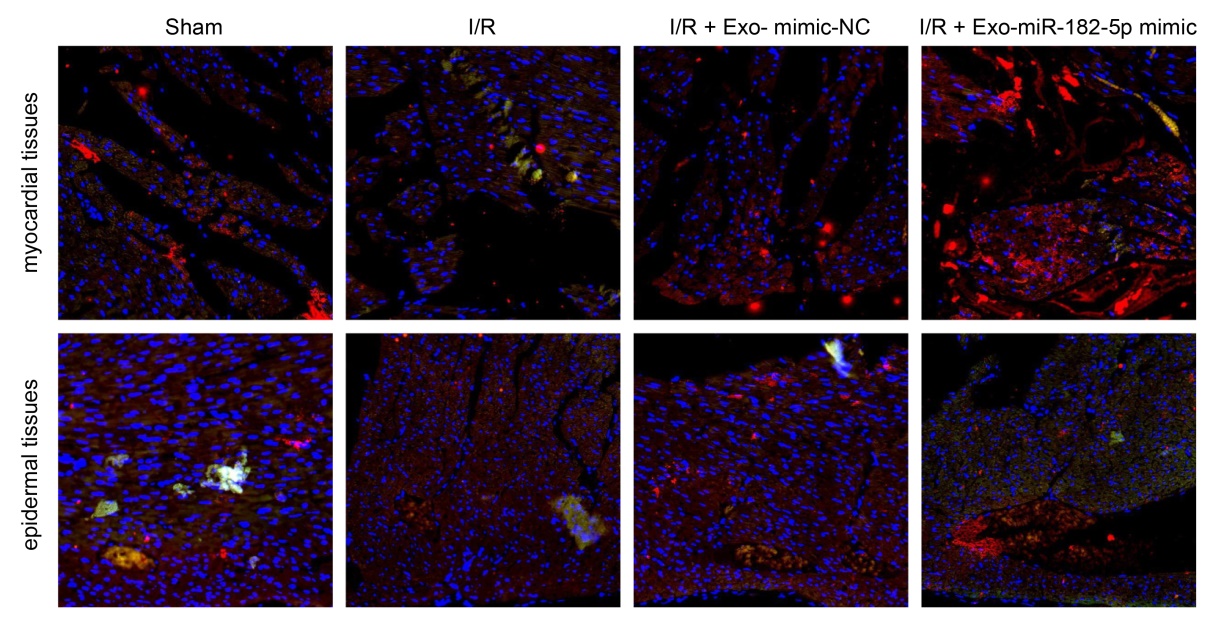
**

**Supplementary Figure 6** miR-182-5p expression in mouse myocardial tissues and epidermal tissues determined by RNA-FISH assay. Red indicates PKH26-labeled exosomes, blue indicates DAPI staining and green indicates cTnI. Each experiment was repeated three times independently.

**
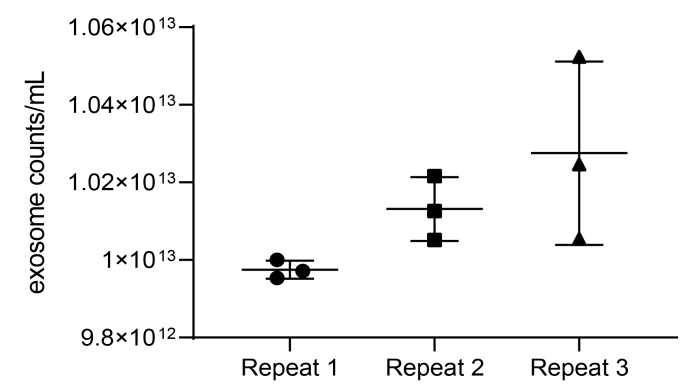
**

**Supplementary Figure 7** Contents of exosomes derived from the cultured 1 × 10^7^ MSCs determined by NFCM/NanoFCM Each experiment was repeated three times independently.
