# Supplementary material for: Mesenchymal stem cell-derived exosomal microRNA-182-5p alleviates myocardial ischemia/reperfusion injury by targeting GSDMD in mice
Source: Cell Death Discov. 2022 Apr 14;8:202. doi: 10.1038/s41420-022-00909-6 (PMC9010441; doi:10.1038/s41420-022-00909-6)
Supplement: Supplementary file 2 — Supplementary Tables [file 41420_2022_909_MOESM2_ESM.docx]

**Supplementary Table 1** The cardiac function parameters in I/R and sham-operated mice.

| Indexes | Groups | |
| --- | --- | --- |
|  | I/R | Sham |
| LVEF | 57.24 ± 13.84% | 79.64 ± 5.59% |
| LVFS | 25.42 ± 8.52% | 41.53 ± 5.04% |
| LVESD | 0.96 ± 0.23 mm | 0.52 ± 0.08 mm |
| LVEDD | 1.28 ± 0.23 mm | 0.90 ± 0.18 mm |

Note: I/R, ischemia reperfusion; LVEF, left ventricular ejection fraction; LVFS, left ventricular fractional shortening; LVESD, left ventricular end systolic diameter; LVEDD, left ventricular end diastolic diameter.

**Supplementary Table 2** Primer sequences for RT-qPCR

| Gene | Sequence |
| --- | --- |
| miR-182-5p | F: 5'-GGCAATGGTAGAACTCACAC'-3' |
|  | R: Reverse universal primer |
| GSDMD | F: 5'-ACTGAGGTCCACAGCCAAGAGG'-3' |
|  | R: 5'-GCCACTCGGAATGCCAGGATG'-3' |
| GAPDH | F: 5'-TTCACCACCATGGAGAAGGC'-3' |
|  | R: 5'-GGCATGGACTGTGGTCATGA'-3' |
| U6 | F: 5'-CTCGCTTCGGCAGCACA'-3' |
|  | R: Reverse universal primer |

Note: RT-qPCR, reverse transcription quantitative polymerase chain reaction; miR-182-5p, microRNA-182-5p; GSDMD, gasdermin D; GAPDH, glyceraldehyde-3-phosphate dehydrogenase; F, forward; R, reverse.
