# Supplementary material for: Mesenchymal stem cell-derived exosomal microRNA-182-5p alleviates myocardial ischemia/reperfusion injury by targeting GSDMD in mice
Source: Cell Death Discov. 2022 Apr 14;8:202. doi: 10.1038/s41420-022-00909-6 (PMC9010441; doi:10.1038/s41420-022-00909-6)
Supplement: Supplementary file 3 — Original Western Blots [file 41420_2022_909_MOESM3_ESM.docx]

**Original Western Blots**

Figure 1A


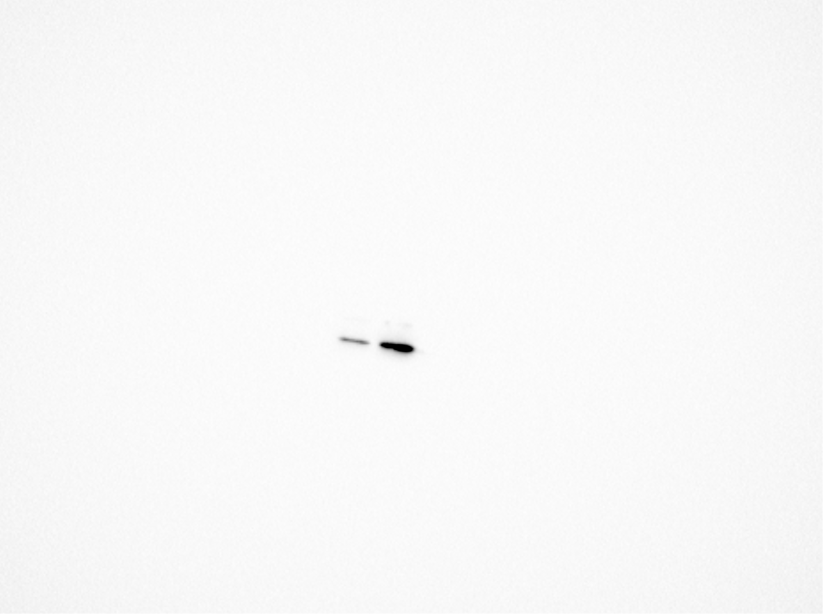
 NLRP3


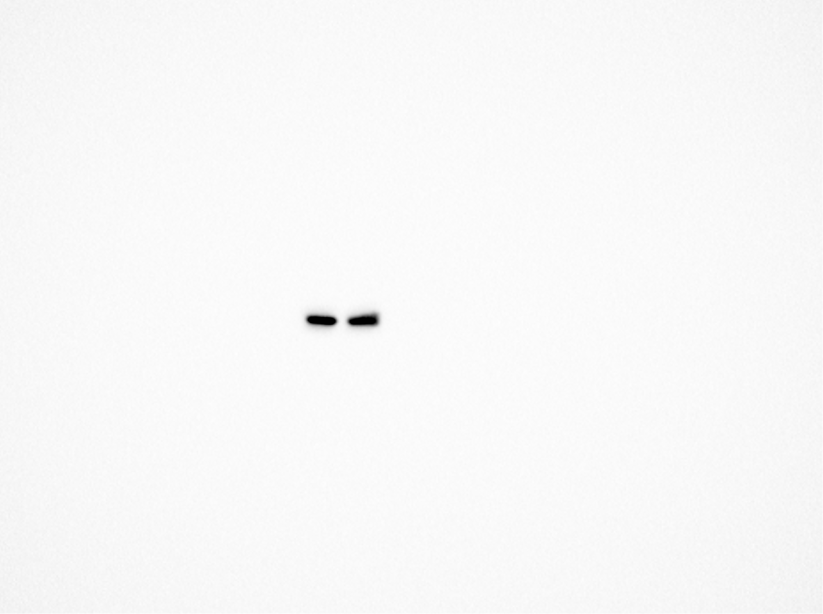
 GAPDH

Figure 1I


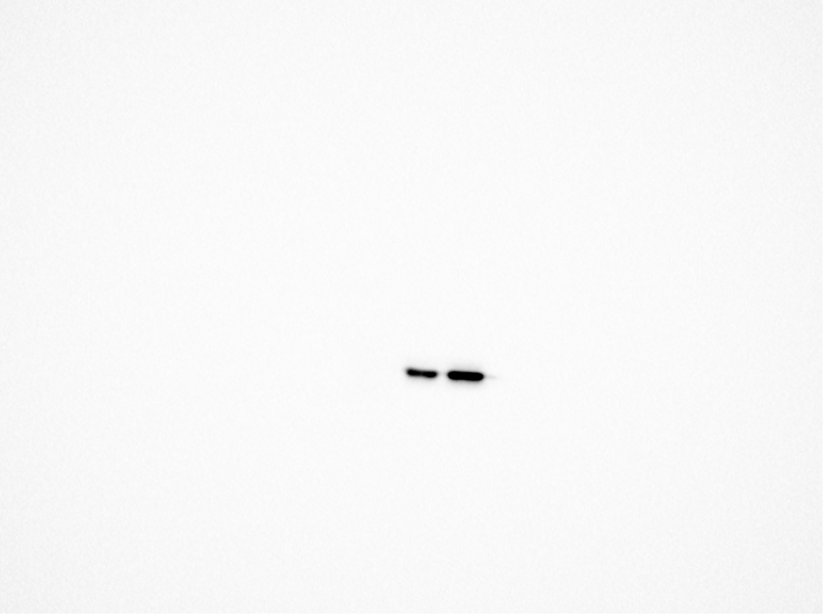
 ASC


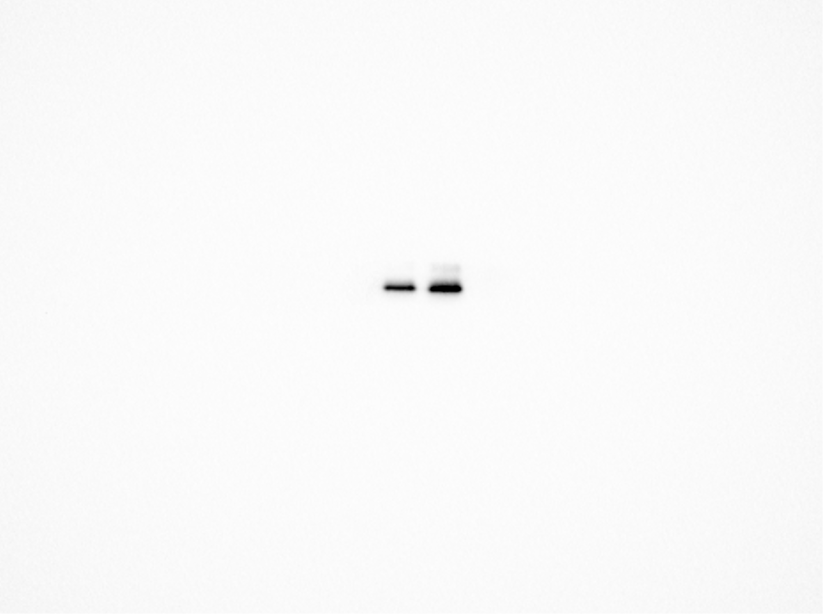
 pro-caspase-1


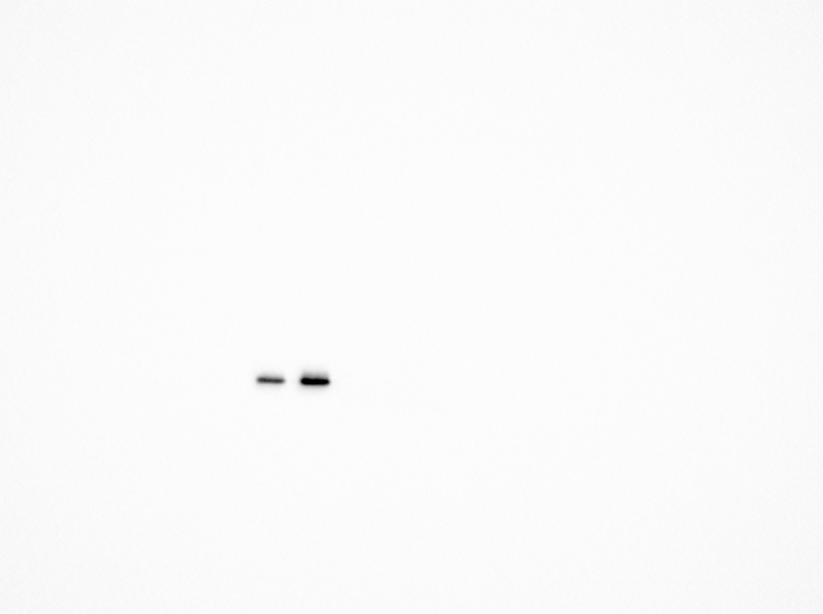
 Cleaved caspase-1


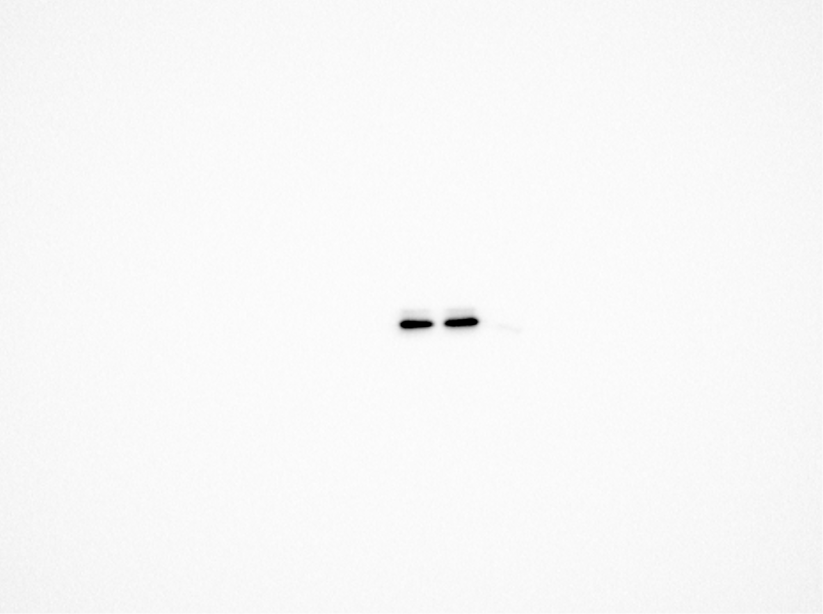
 GAPDH

Figure 2B


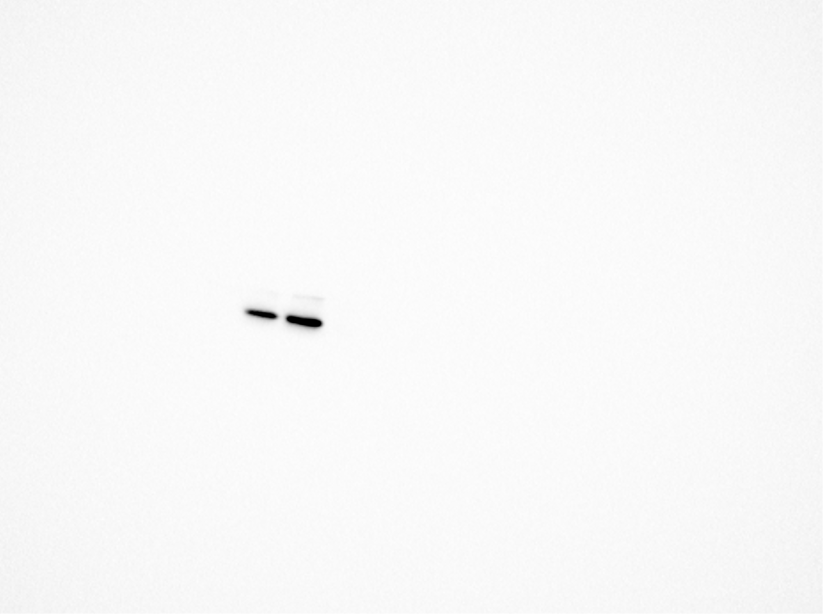
 GSDMD


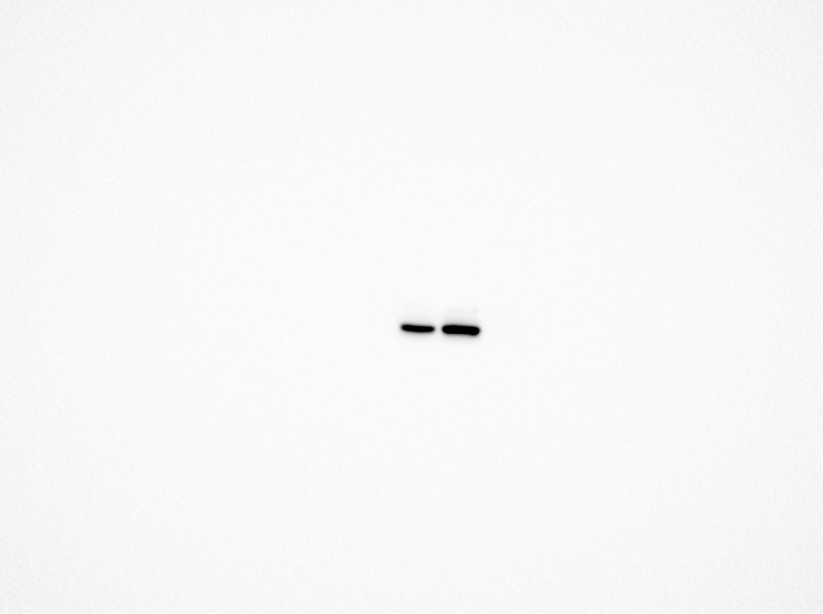
 GSDMD-N


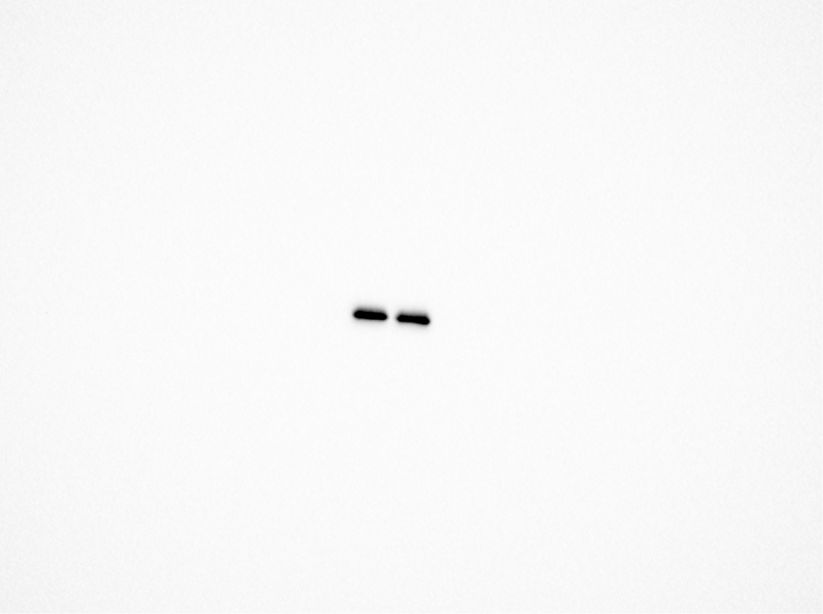
 GAPDH

Figure 4G


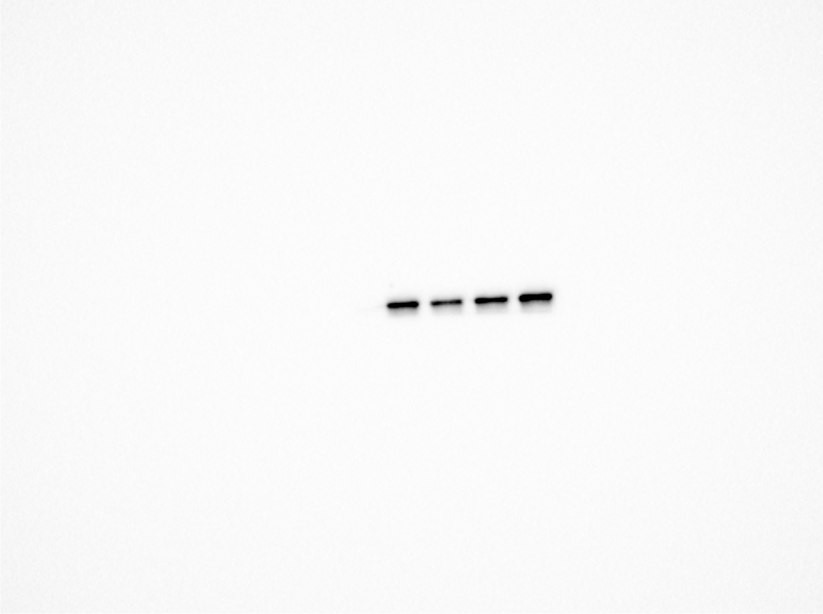
 GSDMD


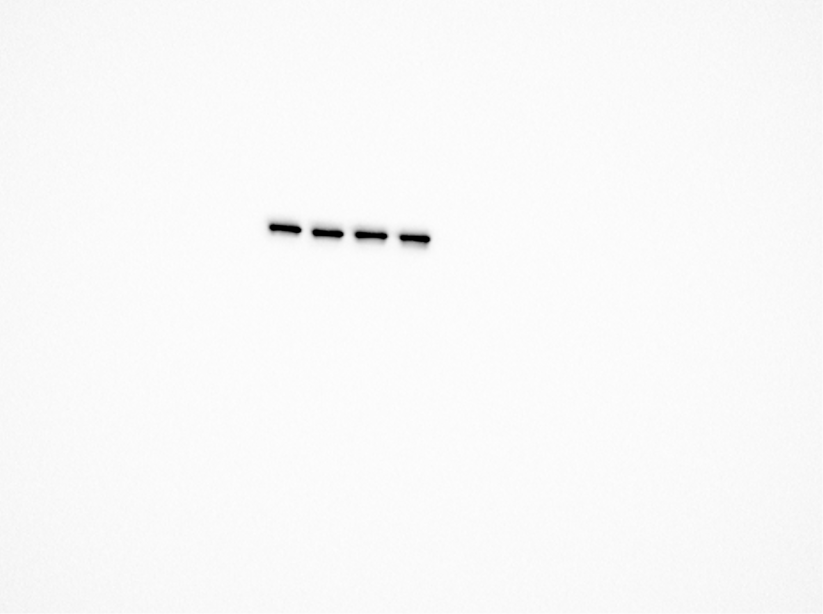
 GAPDH

Figure 7M


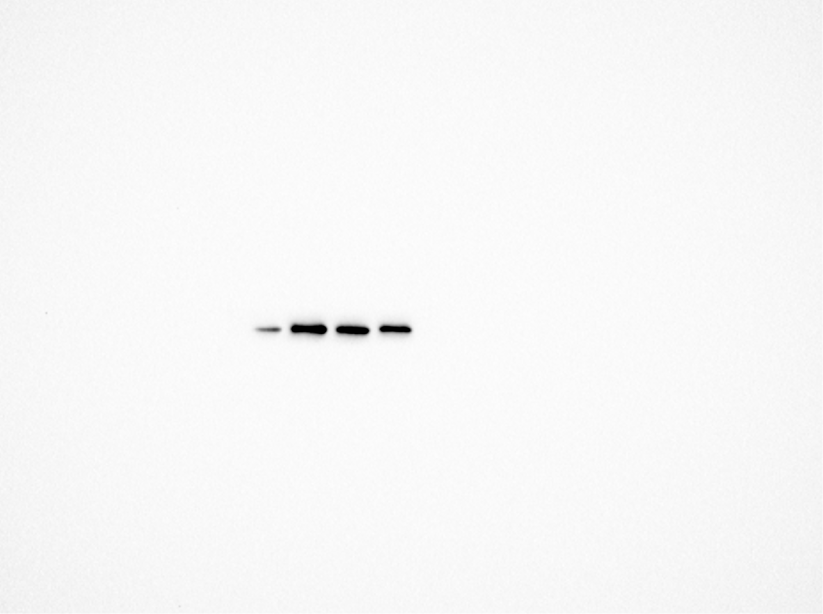
 ASC


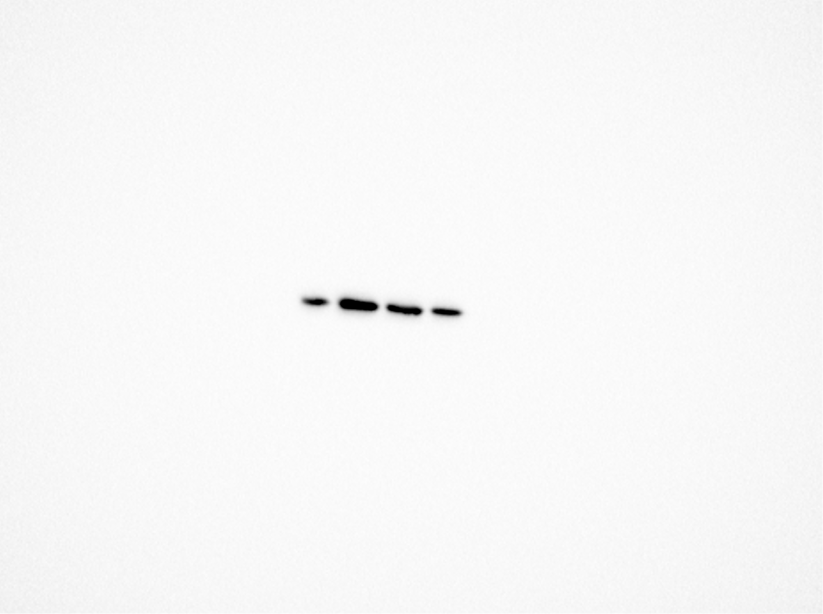
 pro-caspase-1


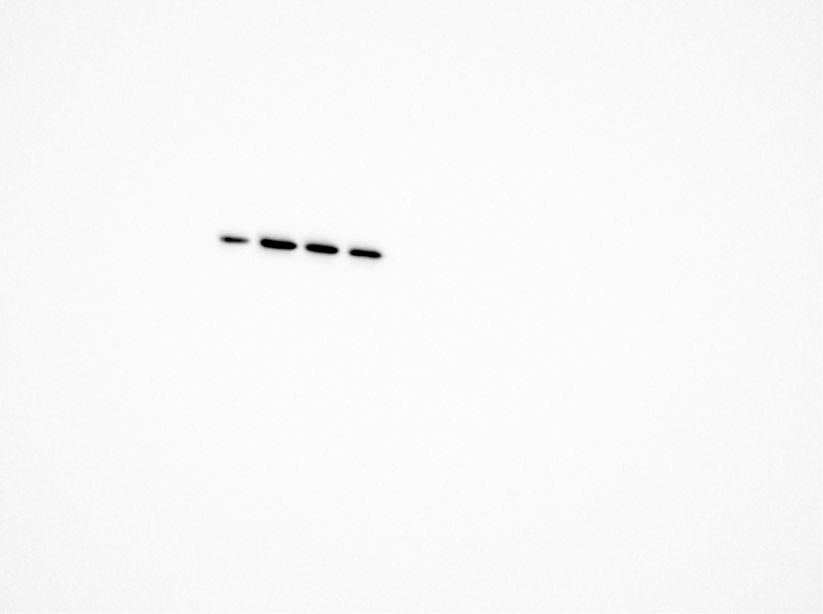
 Cleaved caspase-1


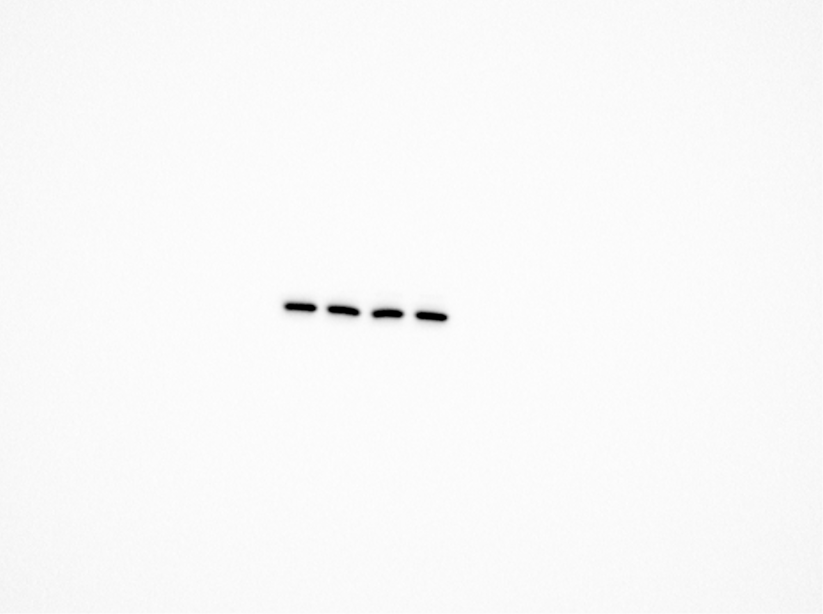
 GAPDH

Supplementary Figure 2


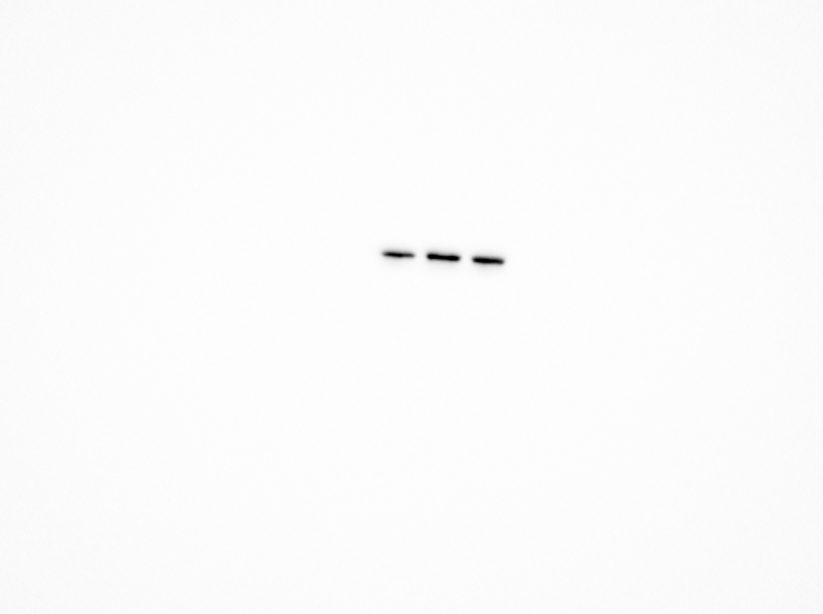
 GSDMD-N


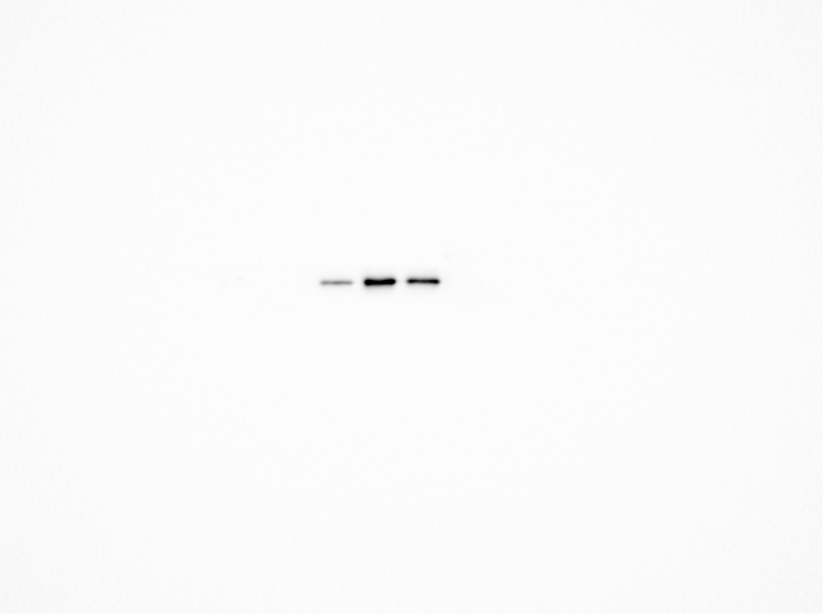
 Cleaved caspase-1


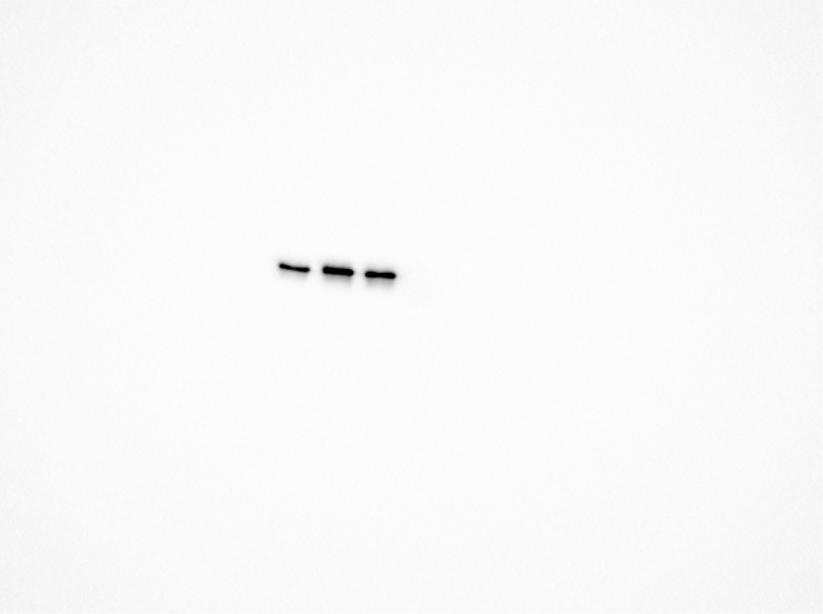
 IL-1β


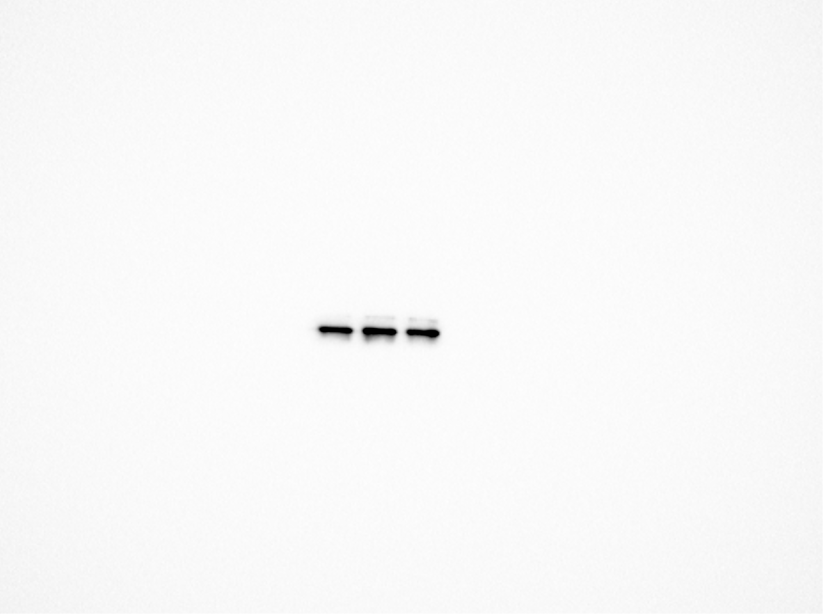
 IL-18


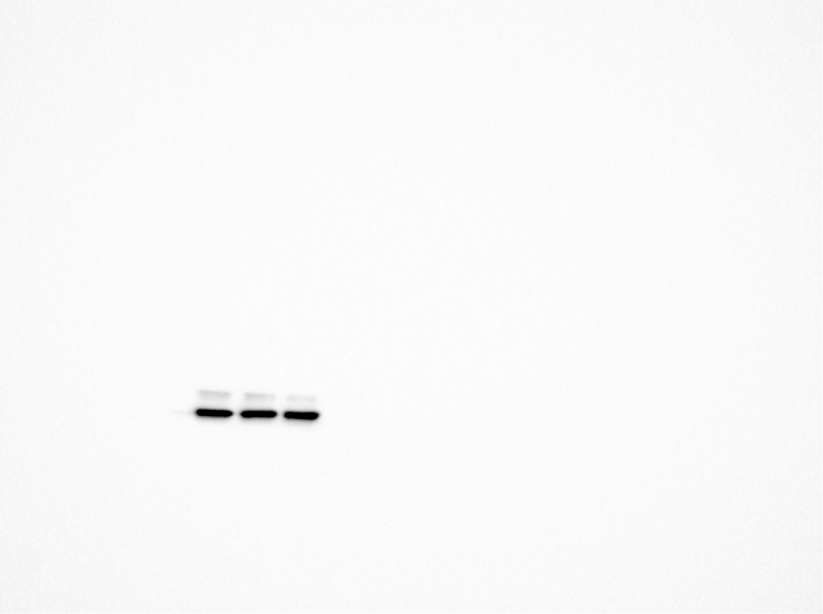
 GAPDH

Supplementary Figure 3F


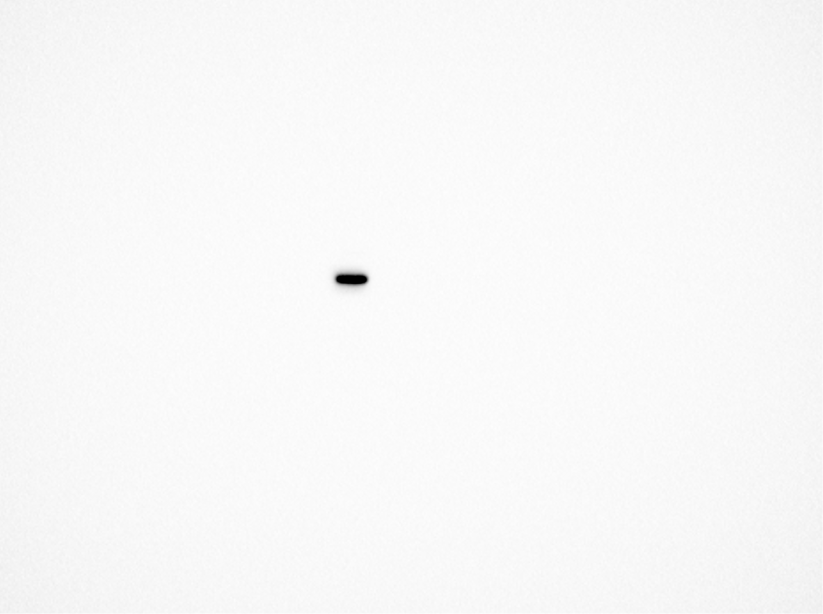
 HSP70


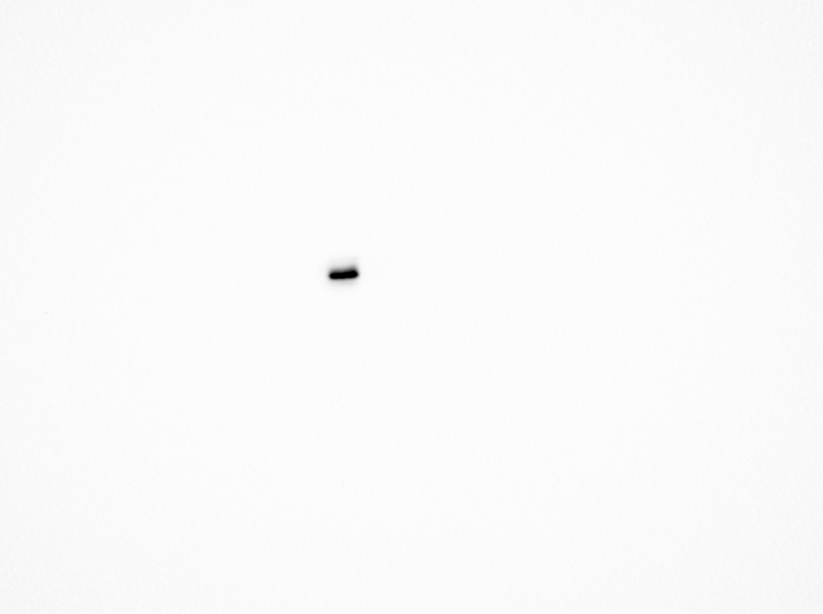
 CD63


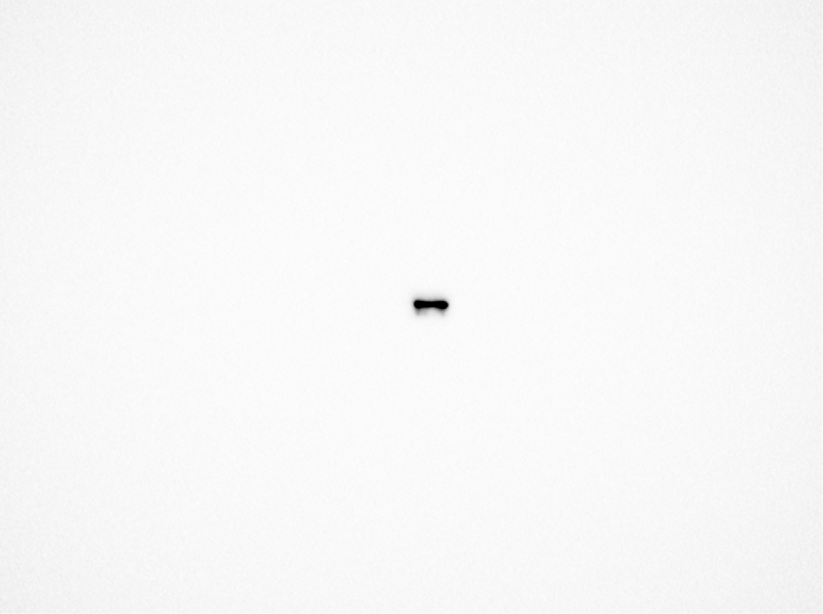
 TSG101


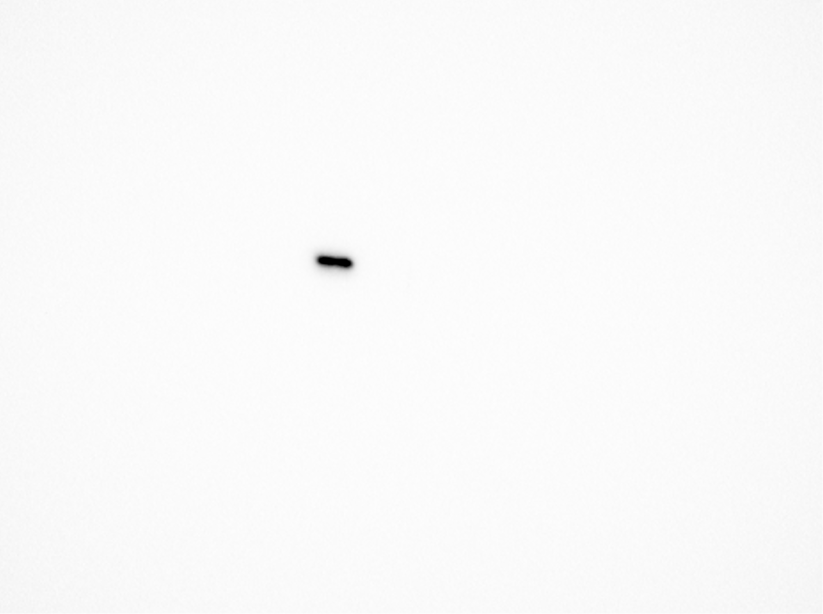
 Alix


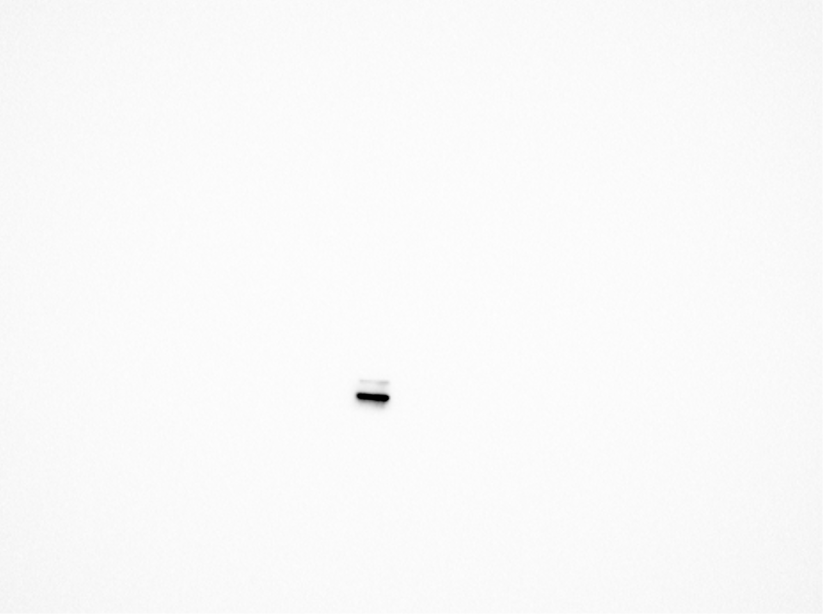
 Calnexin
